# Context-Dependent Functions of NANOG Phosphorylation in Pluripotency and Reprogramming

**DOI:** 10.1016/j.stemcr.2017.03.023

**Published:** 2017-04-27

**Authors:** Arven Saunders, Dan Li, Francesco Faiola, Xin Huang, Miguel Fidalgo, Diana Guallar, Junjun Ding, Fan Yang, Yang Xu, Hongwei Zhou, Jianlong Wang

**Affiliations:** 1 The Black Family Stem Cell Institute, Icahn School of Medicine at Mount Sinai, New York, NY 10029, USA; 2 The Graduate School of Biomedical Sciences, Icahn School of Medicine at Mount Sinai, New York, NY 10029, USA; 3 Department of Cell, Developmental, and Regenerative Biology, Icahn School of Medicine at Mount Sinai, New York, NY 10029, USA; 4 Division of Biological Sciences, University of California, San Diego, La Jolla, CA 92093, USA

**Keywords:** post-translational modification, self-renewal, iPSC, phospho-NANOG, serine to alanine mutation, pluripotency, reprogramming

## Abstract

The core pluripotency transcription factor NANOG is critical for embryonic stem cell (ESC) self-renewal and somatic cell reprogramming. Although NANOG is phosphorylated at multiple residues, the role of NANOG phosphorylation in ESC self-renewal is incompletely understood, and no information exists regarding its functions during reprogramming. Here we report our findings that NANOG phosphorylation is beneficial, although nonessential, for ESC self-renewal, and that loss of phosphorylation enhances NANOG activity in reprogramming. Mutation of serine 65 in NANOG to alanine (S65A) alone has the most significant impact on increasing NANOG reprogramming capacity. Mechanistically, we find that pluripotency regulators (ESRRB, OCT4, SALL4, DAX1, and TET1) are transcriptionally primed and preferentially associated with NANOG S65A at the protein level due to presumed structural alterations in the N-terminal domain of NANOG. These results demonstrate that a single phosphorylation site serves as a critical interface for controlling context-dependent NANOG functions in pluripotency and reprogramming.

## Highlights

• NANOG phospho-residues are evolutionarily conserved in mammals
• Phosphorylation promotes NANOG function in sustaining ESC self-renewal
• Loss of phosphorylation improves NANOG function in reprogramming
• Pluripotency regulators are preferentially associated with NANOG S65A in pre-iPSCs

## Graphical Abstract

**Figure:**
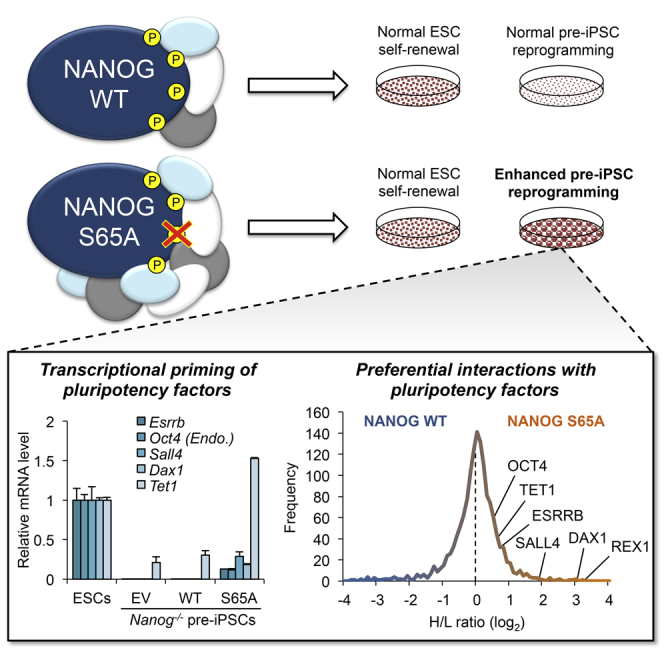

## Teaser

Wang and colleagues outline the role of phosphorylation in regulating NANOG function in ESCs and during reprogramming. Phosphorylation promotes NANOG function in maintaining ESC self-renewal, whereas loss of phosphorylation increases NANOG activity in reprogramming. Mutation of NANOG S65 results in transcriptional priming of pluripotency genes and their preferential association with NANOG S65A at the protein level, resulting in increased reprogramming efficiency.

## Introduction

The phospho-proteome in pluripotent stem cells has been extensively and systematically studied (Van Hoof et al., 2012), and has uncovered phosphorylated residues on pluripotency factors that play important roles in establishing and maintaining pluripotency. Despite the fact that NANOG was speculated to be a phospho-protein over a decade ago (Yates and Chambers, 2005), very little information is available regarding the status and functional implications of NANOG phosphorylation. Studies coupling immunoprecipitation with mass spectrometry (IP-MS) have found that human NANOG is phosphorylated at 11 different sites by ERK2 and CDK1 in human embryonic stem cells (ESCs) (Brumbaugh et al., 2014), and that mouse NANOG is phosphorylated at four different sites (Li et al., 2011, Moretto-Zita et al., 2010) by ERK1 as well as unidentified kinases (Kim et al., 2014). The specific role of phosphorylation in regulating NANOG function, however, remains elusive.

One study suggested that phosphorylation is important for maintaining NANOG stability in ESCs (Moretto-Zita et al., 2010). This study relied on ectopic expression of NANOG in HEK293 cells for identification of phosphorylation sites by IP-MS, and tested the functions of these phosphorylation sites with phospho-dead or phospho-mimic mutants in the presence of endogenous NANOG in wild-type (WT) mouse ESCs (mESCs) (Moretto-Zita et al., 2010). In contrast, another study reported that phosphorylation of NANOG by ERK1 during differentiation of ESCs decreases NANOG stability through ubiquitination-mediated degradation (Kim et al., 2014). Here we systematically investigated the function of NANOG phosphorylation in two biological settings within a physiological context where NANOG function is critical and endogenous NANOG interference with phospho-dead and phospho-mimic mutants is minimized. Our findings therefore contribute important functional data to the phospho-proteome in pluripotent stem cells, and improve our understanding of the key pluripotency regulator NANOG in controlling ESC pluripotency and somatic cell reprogramming.

## Results

### NANOG Is Phosphorylated at Ser56/57 and Ser65 in mESCs

**Figure 1:**
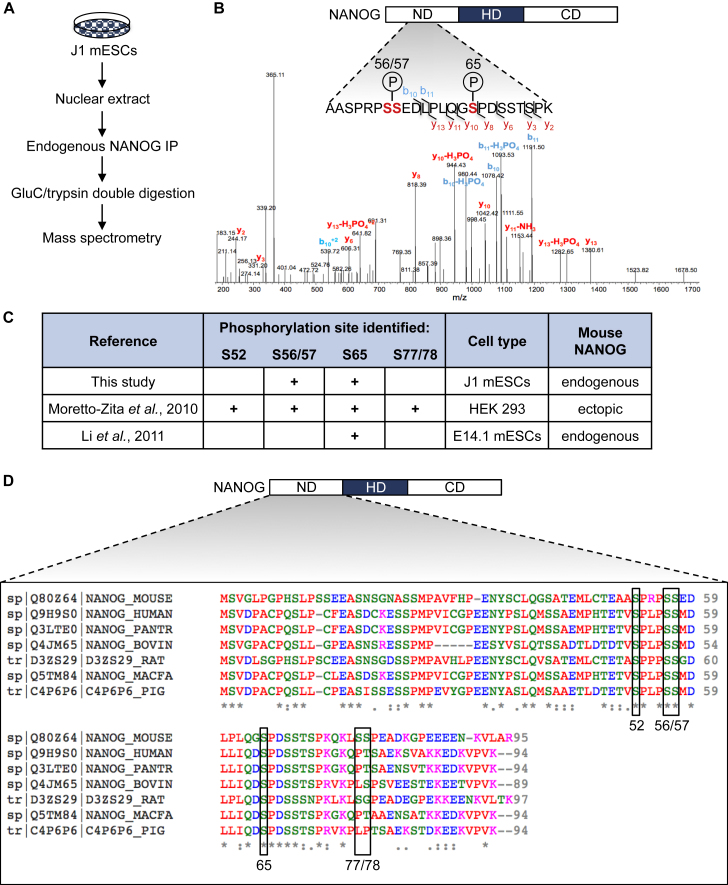
Identification of Phosphorylated Residues on Endogenous NANOG in ESCs (A) Experimental design for endogenous NANOG IP-MS in J1 mESCs. (B) Annotated spectrum identifying S56/57 and S65 as phosphorylated residues in the N terminus of NANOG. ND, N-terminal domain; HD, homeodomain; CD, C-terminal domain. (C) Summary of all studies to date that have identified phosphorylated residues on mouse NANOG by IP-MS. (D) Sequence alignment of the N-terminal domains of mouse, human, chimpanzee, bovine, rat, macaque, and pig NANOG (from top to bottom). Phospho-residues identified in mouse NANOG by all IP-MS studies are boxed. Asterisks indicate full amino acid conservation and colons indicate partial conservation. ND, N-terminal domain; HD, homeodomain; CD, C-terminal domain.

We performed IP-MS of endogenous NANOG in J1 mESCs (Figure 1A), and identified S56/57 and S65 as phosphorylated residues in the N terminus of NANOG (Figure 1B). We were unable, however, to distinguish phosphorylation between adjacent residues S56 and S57, similar to what others reported (Moretto-Zita et al., 2010). Comparison of our NANOG IP-MS analysis with that of other groups (Li et al., 2011, Moretto-Zita et al., 2010) revealed S65 as the only mouse NANOG phosphorylation site consistently identified by all studies to date, suggesting potential importance for this residue in regulating NANOG function (Figure 1C). Interestingly, multiple sequence alignment of NANOG N-terminal domains revealed the full conservation of serines 52, 56/57, and 65 across several mammalian species (Figure 1D), suggesting that these phospho-sites may be evolutionarily conserved to maintain NANOG function. In support of this hypothesis, human NANOG is also phosphorylated at S52, S56/57, and S65 in human ESCs (Brumbaugh et al., 2014), although the functions of these modifications remain undefined.

### Phosphorylation Promotes NANOG Function in Sustaining mESC Self-Renewal

**Figure 2:**
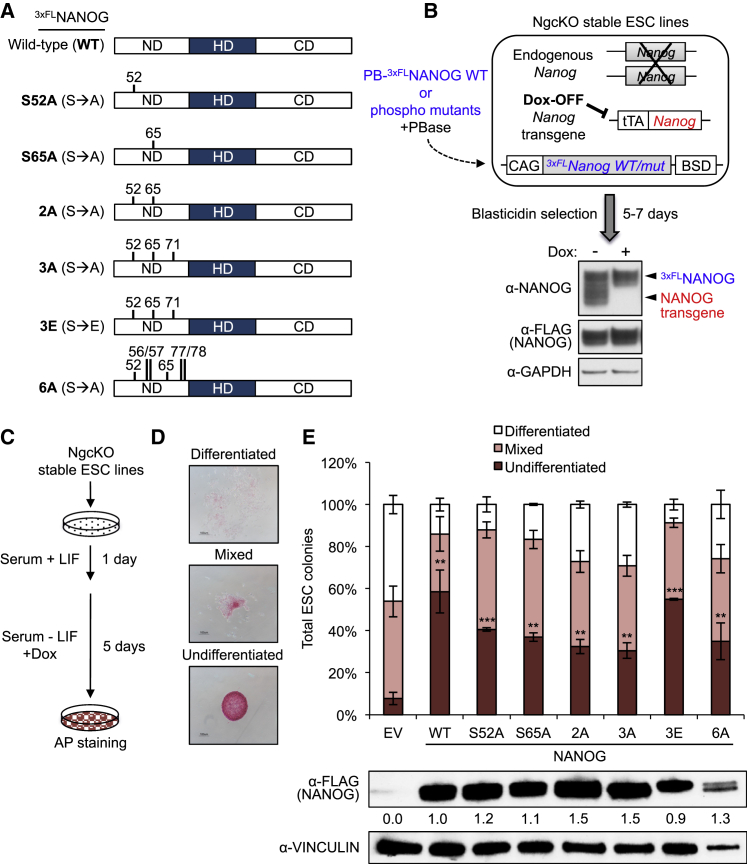
Phosphorylation Is Dispensable for NANOG to Sustain LIF-Independent mESC Self-Renewal (A) 3xFLAG-tagged NANOG phospho-mutants used in this study. S→A denotes serine to alanine mutation and S→E serine-to-glutamic acid mutation of the indicated residues. ND, N-terminal domain; HD, homeodomain; CD, C-terminal domain. (B) Schematic of the generation of *Nanog* conditional knockout (NgcKO) ESC lines stably expressing PiggyBac (PB) NANOG phospho-mutants. Western blot demonstrates the complete absence of the *Nanog* transgene after 24 hr of doxycycline (Dox; 1 μg/mL) treatment. (C) Experimental design of the LIF withdrawal colony-formation assay. (D) Representative images of ESC colony morphologies scored after AP staining. (E) Phosphorylation is dispensable for NANOG to maintain ESC self-renewal. Data are presented as average percentages ± SD (n = 3 independent experiments; ^∗∗^p < 0.01, ^∗∗∗^p < 0.001). Statistical significances are relative to EV control. Western blots were performed on whole-cell lysates from stable ESC lines grown in serum + LIF conditions plus 24 hr of Dox (1 μg/mL) treatment.

To comprehensively test the functions of all previously identified NANOG phosphorylation sites in the maintenance of pluripotency, we investigated how NANOG phospho-dead (S52A, S65A, 2A, 3A, and 6A) and phospho-mimic (3E) mutants (Figure 2A) could maintain pluripotency of ESCs in a *Nanog*^*−/−*^ setting. We utilized doxycycline (Dox)-inducible *Nanog* conditional knockout (NgcKO) ESCs (Das et al., 2011) to generate stable cell lines overexpressing these NANOG phospho-mutants (Figure 2B) and tested the extent to which NANOG phospho-mutants could rescue leukemia inhibitory factor (LIF)-independent self-renewal in NgcKO ESCs.

We seeded NgcKO ESCs at clonal density, withdrew LIF, and added Dox for 5 days, then performed alkaline phosphatase (AP) staining to assess pluripotency status based on colony morphology (Figures 2C and 2D). As expected, cells expressing empty vector (EV) control generated only ∼8% undifferentiated colonies out of the total number of colonies scored after 5 days of LIF withdrawal combined with Dox treatment (Figure 2E). We also noticed that phospho-dead mutants were less efficient than WT NANOG in rescuing ESC self-renewal upon LIF withdrawal. Notably, phospho-dead NANOG 3A was less efficient than its corresponding phospho-mimic NANOG 3E in forming undifferentiated colonies (Figure 2E), supporting that phosphorylation is beneficial to NANOG function in maintaining ESCs. Interestingly, however, we found that all NANOG phospho-mutants could sustain LIF-independent self-renewal significantly better than EV control (Figure 2E), indicating that phosphorylation is not essential for NANOG to maintain self-renewal of ESCs.

### Blocking Phosphorylation at Ser65 Enhances NANOG Reprogramming Activity

**Figure 3:**
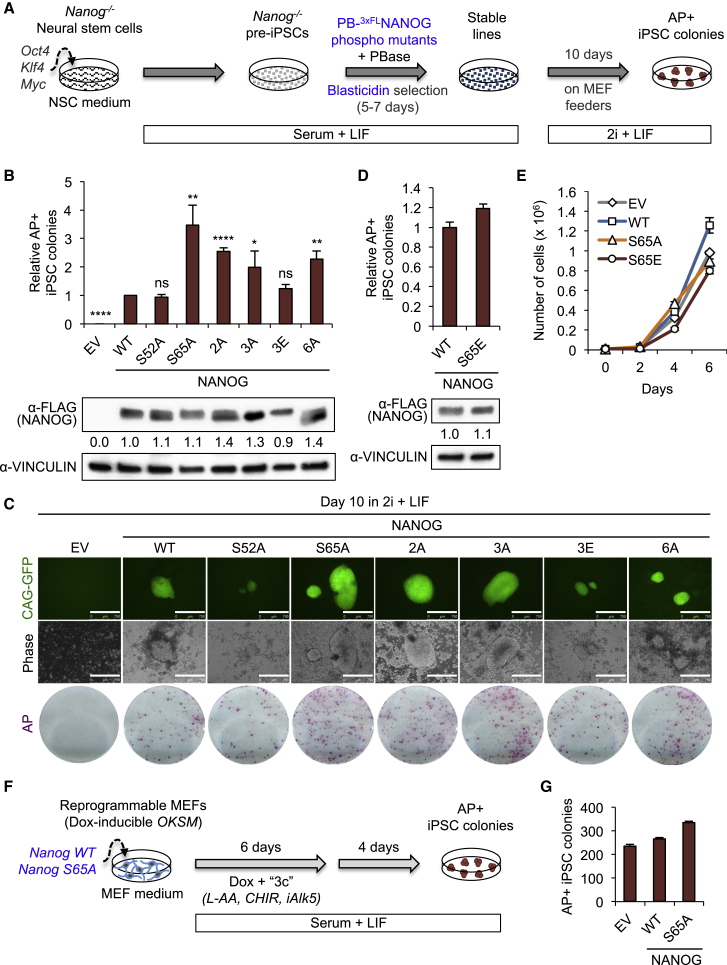
Loss of Phosphorylation Significantly Enhances NANOG Activity in Reprogramming (A) The *Nanog*^*−/−*^ neural stem cell (NSC)-derived pre-iPSC reprogramming system used for assessing NANOG phosphorylation gain or loss of function (see Experimental Procedures for more information). (B) NANOG phospho-dead mutants are more efficient than NANOG WT in pre-iPSC reprogramming. Data are presented as average fold change of AP + iPSC colonies ± SD (n = 3 independent experiments; ^∗^p < 0.05, ^∗∗^p < 0.01, ^∗∗∗∗^p < 0.0001; ns, not significant). (C) Representative images of CAG-GFP + iPSC colonies as well as whole-well images of AP + iPSC colonies. Scale bars represent 750 μm. (D) NANOG S65E phospho-mimic behaves like NANOG WT in pre-iPSC reprogramming. Data are presented as average fold change of AP + iPSC colonies ±SD (n = 3 technical replicates). (E) Proliferation curve for *Nanog*^*−/−*^ pre-iPSCs expressing EV, NANOG WT, NANOG S65A, and NANOG S65E. (F) The doxycycline (Dox)-inducible MEF reprogramming system. (G) NANOG S65A enhances OKSM-mediated MEF reprogramming. Data are presented as average fold change of AP + iPSC colonies ±SD (n = 3 technical replicates). See also Figure S1.

NANOG is critical for executing the final stage of reprogramming in various contexts (Costa et al., 2013, Silva et al., 2009); however, the role of NANOG phosphorylation in regulating this process has not been explored. We therefore tested how efficiently NANOG phospho-mutants could reprogram partially reprogrammed *Nanog*^*−/−*^ somatic cells (pre-iPSCs) to naive induced pluripotent stem cells (iPSCs) (Figure 3A). As expected, we observed no iPSC colonies generated by pre-iPSCs expressing EV control after 10 days in serum-free, defined medium containing inhibitors of GSK3β and MEK/ERK (“2i + LIF”) (Figure 3B). We also did not observe any significant differences between the numbers of AP + iPSC colonies generated by NANOG WT and either NANOG S52A or NANOG 3E (a phospho-mimic). Surprisingly, however, we found that the NANOG phospho-dead mutants 2A, 3A, 6A, and especially S65A, generated significantly more AP + iPSC colonies than NANOG WT (Figures 3B and 3C). These data indicate that phosphorylation is not required for the ability of NANOG to execute the final stage of reprogramming, and importantly, that loss of phosphorylation significantly improves the capacity for NANOG to drive pre-iPSC reprogramming.

In particular, we found that out of all phospho-mutants tested, the single point mutant NANOG S65A was the most efficient in reprogramming *Nanog*^*−/−*^ pre-iPSCs, reproducibly generating ∼3.5-fold more AP + iPSC colonies than NANOG WT (Figures 3B and S1A). We next generated a phospho-mimic for this residue, NANOG S65E, and found no noticeable difference between the numbers of AP + iPSC colonies generated by either NANOG WT or NANOG S65E (Figures 3D and S1A), strongly suggesting that blocking phosphorylation at S65 can dramatically improve the reprogramming function of NANOG during the pre-iPSC to iPSC transition. Additionally we found that the proliferation rates of pre-iPSCs expressing EV, NANOG WT, NANOG S65A, and NANOG S65E were all comparable (Figure 3E), indicating that the increased activity of NANOG S65A in reprogramming was not due to an increase in proliferation rate.

To determine whether the same enhanced reprogramming activity of NANOG S65A could also be observed in conventional fibroblast reprogramming, we compared the ability of NANOG WT and S65A to enhance OKSM-mediated mouse embryonic fibroblast (MEF) reprogramming (Vidal et al., 2014) (Figure 3F). Addition of either NANOG WT or S65A consistently increased reprogramming efficiency over EV in two independent experiments, although NANOG S65A was appreciably more effective than NANOG WT in enhancing MEF reprogramming (Figures 3G and S1B).

Collectively, these results indicate that phosphorylation is dispensable for NANOG activity in reprogramming, and that blocking phosphorylation at S65 can enhance NANOG reprogramming activity.

### S65A Mutation Does Not Affect NANOG Protein Stability or Subcellular Distribution

**Figure 4:**
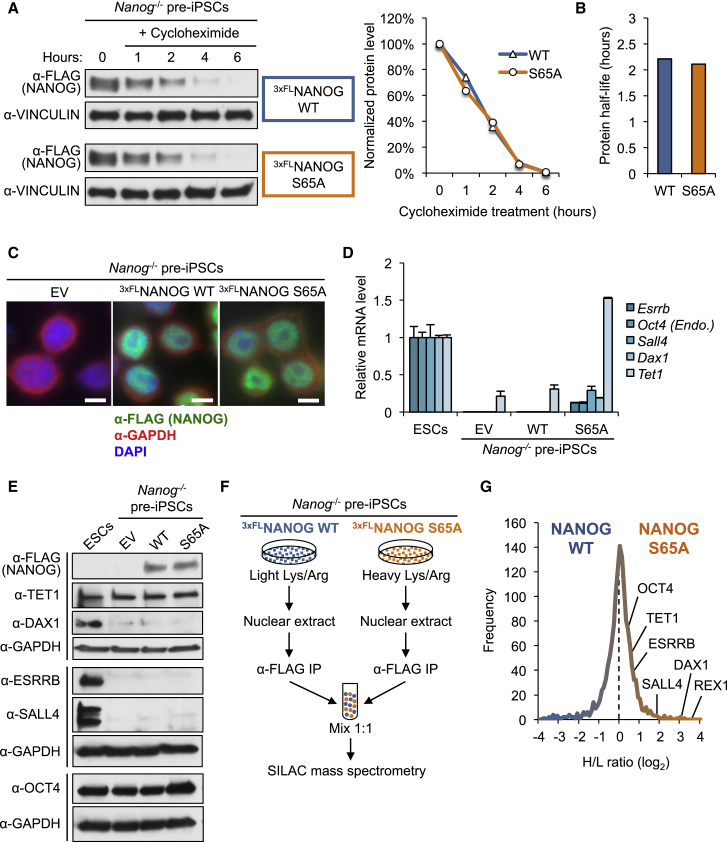
Pluripotency Factors Are Preferentially Associated with NANOG S65A Pre-iPSCs (A) Western blot analysis of a 6-hr cycloheximide (50 μg/mL) time-course treatment in *Nanog*^*−/−*^ pre-iPSCs expressing ^3xFLAG^NANOG WT or ^3xFLAG^NANOG S65A in serum + LIF culture conditions. (B) Half-lives of NANOG WT and NANOG S65A were calculated using linear regression analysis of the data plotted in (A). (C) Immunofluorescence for ^3xFLAG^NANOG WT and S65A in pre-iPSCs. GAPDH and DAPI were used as cytoplasmic and nuclear markers, respectively. Scale bars represent 5 μm. (D) RT-PCR analysis for *Esrrb*, endogenous (*Oct4* Endo.), *Sall4*, *Dax1*, and *Tet1* mRNA levels in ^3xFLAG^NANOG WT and S65A pre-iPSCs, as well as WT E14T ESCs. RNA was collected from stable pre-iPSC lines immediately following blasticidin selection (see Figure 3A). Data are presented as average ± SD (n = 3 technical replicates), relative to *β-actin* housekeeping control, and each gene is normalized to the levels in E14T mESCs. (E) Western blot analyses of FLAG, TET1, DAX1, ESRRB, SALL4, OCT4, and GAPDH (loading control) protein levels in J1 mESCs, and in pre-iPSCs expressing EV, ^3xFLAG^NANOG WT, and S65A pre-iPSC whole-cell lysates. Total lysates were collected from stable pre-iPSC lines immediately following blasticidin selection (see Figure 3A). (F) Illustration of the SILAC mass spectrometry experiment performed on stable *Nanog*^*−/−*^ pre-iPSCs expressing ^3xFLAG^NANOG WT and S65A. ^3xFLAG^NANOG WT pre-iPSCs were cultured in serum + LIF medium containing light isotope-labeled lysine and arginine, and ^3xFLAG^NANOG S65A pre-iPSCs were cultured in serum + LIF medium containing heavy isotope-labeled lysine and arginine. (G) Histogram of the frequency distribution of heavy/light (H/L) ratios (log_2_ scale) of all proteins identified by SILAC IP-MS. The pluripotency factors REX1, DAX1, SALL4, ESRRB, TET1, and OCT4 were identified as putative preferential interacting partners of ^3xFLAG^NANOG S65A, compared with ^3xFLAG^NANOG WT, in pre-iPSCs. See also Figure S2.

To determine whether the increased reprogramming activity of S65A over WT NANOG was due to enhanced protein stability, we treated NANOG WT and NANOG S65A pre-iPSCs with the translation inhibitor cycloheximide over a 6-hour time course. We did not observe any differences in the degradation rates or the protein half-lives of NANOG WT and NANOG S65A (Figures 4A and 4B), indicating that the enhanced reprogramming activity of NANOG S65A was not due to increased protein stability.

Next, we performed immunofluorescence for NANOG WT and NANOG S65A in pre-iPSCs and found that both were predominantly nuclear, with no obvious differences in overall distribution (Figure 4C), which we also confirmed by subcellular protein fractionation (Figure S2A).

Taken together, we conclude that the enhanced reprogramming activity of NANOG S65A is not due to increased protein stability or altered subcellular distribution.

### Pluripotency Regulators Are Preferentially Associated with NANOG S65A in Pre-iPSCs

NANOG has been shown to prime the expression of certain pluripotency genes, such as *Esrrb* and *Oct4* in pre-iPSCs, to promote reprogramming efficiency (Costa et al., 2013). Using a candidate approach, we tested whether NANOG S65A overexpression affected the expression levels of genes known to be critical for the reprogramming process. We found that the pluripotency-associated genes *Esrrb*, *Oct4*, *Sall4*, *Dax1*, and *Tet1* were all transcriptionally upregulated in NANOG S65A pre-iPSCs, compared with NANOG WT pre-iPSCs, although still far below their ESC levels (except for *Tet1*) (Figure 4D). Western blotting of whole-cell lysates revealed that, relative to their protein expression in ESCs, these pluripotency regulators were either non-detectable (e.g., DAX1, ESRRB, and SALL4) or equally abundant in NANOG WT and S65A pre-iPSCs when expressed at detectable levels (e.g., TET1 and OCT4) (Figure 4E), suggesting a potential post-transcriptional regulation of these pluripotency gene transcripts.

NANOG can physically interact and synergize with pluripotency regulators such as TET1 to promote pre-iPSC reprogramming (Costa et al., 2013). To overcome the western detection threshold for physical associations of NANOG WT and S65A with those transcriptionally activated pluripotency regulators in pre-iPSCs, we utilized a more sensitive approach, namely SILAC (stable isotope labeling by amino acids in cell culture)-based quantitative mass spectrometry, coupled with IP of NANOG to compare the WT and S65A NANOG interactomes in *Nanog*^*−/−*^ pre-iPSCs (Figure 4F). As expected, the majority of proteins identified by SILAC IP-MS were equally abundant in both cell populations (heavy/light log_2_ ratio ≈ 0). Most importantly, however, we detected a preferential enrichment of all the pluripotency regulators tested in Figures 4E and 4F (TET1, DAX1, ESRRB, SALL4, and OCT4) in the NANOG S65A interactome compared with the NANOG WT interactome (Figures 4G and S2B).

Together, we conclude that loss of phosphorylation may endow NANOG S65A with increased affinity with other pluripotency-associated regulators, leading to enhanced reprogramming (see Discussion and Figure S2D).

## Discussion

Here we report our findings on the role of NANOG phosphorylation in the maintenance and establishment of pluripotency. While phosphorylation was not essential for NANOG to maintain pluripotency of ESCs, it seemed to be beneficial for NANOG to sustain ESC self-renewal. Conversely, however, we found that loss of phosphorylation promoted NANOG function in reprogramming.

What might be the underlying cause of such context-dependent functions of NANOG phosphorylation in pluripotency and reprogramming? We did not observe any differences in protein stability or subcellular localization between NANOG WT and S65A in *Nanog*^*−/−*^ pre-iPSCs (Figure 4). The intrinsic transcriptional activity also does not seem to be affected in S65A mutant relative to WT NANOG, as we observed no noticeable difference in the abilities of WT and S65A NANOG to activate a *Nanog* enhancer-driven luciferase reporter (Figure S2C). Importantly, our highly sensitive SILAC IP-MS studies indicated that the NANOG S65A interactome in pre-iPSCs is preferentially enriched for the pluripotency factors ESRRB, OCT4, SALL4, DAX1, and TET1, compared with the WT NANOG interactome, in pre-iPSCs (Figure 4G). These factors often co-occupy ESC super-enhancers with NANOG, and have all been implicated in the reprogramming process (Costa et al., 2013, Huang and Wang, 2014). Therefore, it is highly likely that, despite minimal expression levels of DAX1, ESRRB, and SALL4, or equal abundance of TET1 and OCT4, in pre-iPSCs relative to ESCs (Figure 4E), these pluripotency factors may have a higher affinity with S65A than WT NANOG in forming active transcriptional regulatory complexes to mediate enhanced reprogramming.

Is there a structural implication for such preferential association of NANOG S65A with pluripotency regulators? By applying automated protein structure prediction and modeling for full-length NANOG WT and NANOG S65A using the I-TASSER platform (Roy et al., 2010), we observed an apparent unfolding of the N-terminal domain of S65A compared with WT NANOG (Figure S2D). The N-terminal domain has been shown to be dispensable for NANOG nuclear localization, and has been proposed to serve as an interface for interaction with co-factors important for transcriptional activities of NANOG in maintaining ESC self-renewal (Chang et al., 2009, Guo et al., 2009). Therefore, loss of phosphorylation may have endowed NANOG S65A with an altered structure more conducive to association with those nuclear pluripotency regulators, leading to functional activation of the pluripotency program in reprogramming. Future studies applying X-ray crystallography to solve the full-length WT and S65A NANOG protein structures are warranted to confirm this hypothesis, which is currently a challenge in the field (Hayashi et al., 2015, Jauch et al., 2008). Alternatively, the preferential associations of S65A NANOG with these pluripotency regulators (Figure 4G) may also be due to their subtle increased protein levels that can only be detected by quantitative SILAC IP-MS we have employed. Of note, we found that the total OCT4 protein level in S65A pre-iPSCs was appreciably higher than that in WT pre-iPSCs (Figure 4E), likely resulting from endogenous *Oct4* reactivation.

Which kinase could be responsible for S65 phosphorylation? Mouse NANOG has so far only been found to be phosphorylated by ERK1 in differentiating mESCs at serine residues other than S65, resulting in reduced NANOG protein stability (Kim et al., 2014). Such reported destabilizing effects of ERK1-mediated phosphorylation at neighboring residues other than S65 is expected to be equally applicable to NANOG WT and S65A, and is consistent with increased NANOG protein levels in ESCs under 2i + LIF culture (Silva et al., 2009). While this may provide an explanation for the identical protein stability between NANOG WT and S65A in pre-iPSCs under serum + LIF culture (Figure 4A), it cannot explain the enhanced reprogramming activity of NANOG S65A over WT under standard 2i + LIF culture that contains a MEK/ERK inhibitor (Silva et al., 2008) (Figure 3). Interestingly, human NANOG has also been shown to be phosphorylated by protein kinase Cε (PKCε) in cancer cells (Bourguignon et al., 2009, Xie et al., 2013). However, we were unable to increase the reprogramming efficiency of NANOG WT to that of NANOG S65A by treating pre-iPSCs with either a PKCε-specific translocation inhibitor (Figures S1C and S1D) or a pan-PKC inhibitor Go6983 (Figures S1C and S1E), suggesting that PKC does not phosphorylate mouse NANOG. Future studies will be needed to identify the specific kinase responsible for S65 phosphorylation, and to address its impact on NANOG function in pluripotency and reprogramming.

## Experimental Procedures

### NANOG Phosphorylation Mutants

NANOG phospho-mutant plasmids were generated as described by Moretto-Zita et al. (2010). These NANOG mutants were then subcloned via PCR into PiggyBac-CAG (PB) transposon vectors containing an N-terminal 3xFLAG epitope tag for stable overexpression. NANOG S65E phospho-mimic was generated via site-directed mutagenesis using the QuikChange Lightning Multi Site-Directed Mutagenesis Kit (Agilent Technologies). Following PCR amplification and subcloning, all phospho-mutant constructs were verified by sequencing.

### Pre-iPSC and MEF Reprogramming

*Nanog*^*−/−*^ neural stem cell (NSC)-derived pre-iPSCs were generated and used for reprogramming as described by Silva et al. (2009). In brief, 1.0 × 10^4^ pre-iPSCs were seeded after selection onto gelatin-coated 12-well plates on top of irradiated MEFs and grown in serum + LIF for 2 days before medium switch to 2i + LIF medium (20 ng/mL LIF, 1 μM PD325901, and 3 μM CHIR99021). On day 10 in 2i + LIF, plates were stained for AP activity and iPSC colonies were counted under bright-field microscopy.

MEF reprogramming was performed as described by Vidal et al. (2014) with some modifications. In brief, 3.0 × 10^4^ reprogrammable MEFs containing a Dox-inducible OKSM cassette were infected with retroviral NANOG WT, NANOG S65A, or EV control. The next day 34,000 infected MEFs/well were seeded on top of a feeder layer of irradiated MEF feeders on a 6-well plate coated with gelatin, in “Dox + 3c”-containing ESC medium. On day 6 the medium was switched to ESC medium without Dox or 3c, and plates were stained for AP activity on day 10.

### SILAC IP-MS to Compare NANOG WT and S65A Interactomes in Pre-iPSCs

SILAC IP-MS was performed as described by Ding et al. (2015) with some modifications. In brief, *Nanog*^*−/−*^ pre-iPSCs expressing ^3xFLAG^NANOG WT or ^3xFLAG^NANOG S65A were each expanded to 8 15-cm dishes after culturing for 2 weeks in SILAC ESC medium supplemented with either light or heavy lysine and arginine. Pre-iPSC nuclear extracts were pre-cleared with Protein G agarose beads rotating overnight at 4°C. The next day, ^3xFLAG^NANOG WT or ^3xFLAG^NANOG S65A were immunoprecipitated, washed, and eluted from α-FLAG beads with a 3xFLAG peptide solution. Eluted protein was then concentrated, quantified, mixed in a 1:1 ratio for each sample, and subjected to SDS-PAGE. Finally, whole lanes were excised from the gel and subjected to quantitative liquid chromatography-tandem mass spectrometry analysis.

## Author Contributions

A.S. designed and performed experiments, analyzed data, and wrote the manuscript; D.L. performed experiments and analyzed data; F.F. and X.H. provided bioinformatics support and technical assistance; M.F., D.G., J.D., F.Y., Y.X., and H.Z. provided technical assistance, reagents, and helpful discussions; J.W. conceived the project, designed the experiments, analyzed data, and prepared and approved the manuscript.

## Supplemental Information

Document S1. Supplemental Experimental Procedures, Figures S1 and S2, and Table S1

Document S2. Article plus Supplemental Information
