# Supplementary material for: Context-Dependent Functions of NANOG Phosphorylation in Pluripotency and Reprogramming
Source: Stem Cell Reports. 2017 Apr 27;8(5):1115–23. doi: 10.1016/j.stemcr.2017.03.023 (PMC5425684; doi:10.1016/j.stemcr.2017.03.023)
Supplement: Document S1. Supplemental Experimental Procedures, Figures S1 and S2, and Table S1 [file mmc1.pdf]

**Stem Cell Reports, Volume *8***

## Supplemental Information

# Context-Dependent Functions of NANOG Phosphorylation in Pluripotency and Reprogramming

**Arven Saunders, Dan Li, Francesco Faiola, Xin Huang, Miguel Fidalgo, Diana Guallar, Junjun Ding, Fan Yang, Yang Xu, Hongwei Zhou, and Jianlong Wang**

## SUPPLEMENTAL FIGURES AND FIGURE LEGENDS

### Figure S1 (related to Figure 3). Loss of phosphorylation enhances NANOG activity in reprogramming

(A) NANOG S65E phospho mimic behaves like NANOG WT in pre-iPSC reprogramming. Data from an independent experiment (biological replicate) are presented as average fold change of AP+ iPSC colonies ± SD (n = 3 technical replicates).

(B) NANOG S65A enhances OKSM-mediated MEF reprogramming. Data are presented as average fold change of AP+ iPSC colonies ± SD (n = 3 technical replicates).

(C) The *Nanog*$^{-/-}$ neural stem cell (NSC)-derived pre-iPSC reprogramming system used for assessing PKC inhibition.

(D) PKCε peptide inhibitor does not affect the enhanced reprogramming capacity of NANOG S65A over WT. Data are presented as average fold change of AP+ iPSC colonies ± SD (n = 3 technical replicates).

(E) Pan-PKC inhibition does not affect the enhanced reprogramming capacity of NANOG S65A over WT. Data are presented as average fold change of AP+ iPSC colonies ± SD (n = 3 technical replicates).

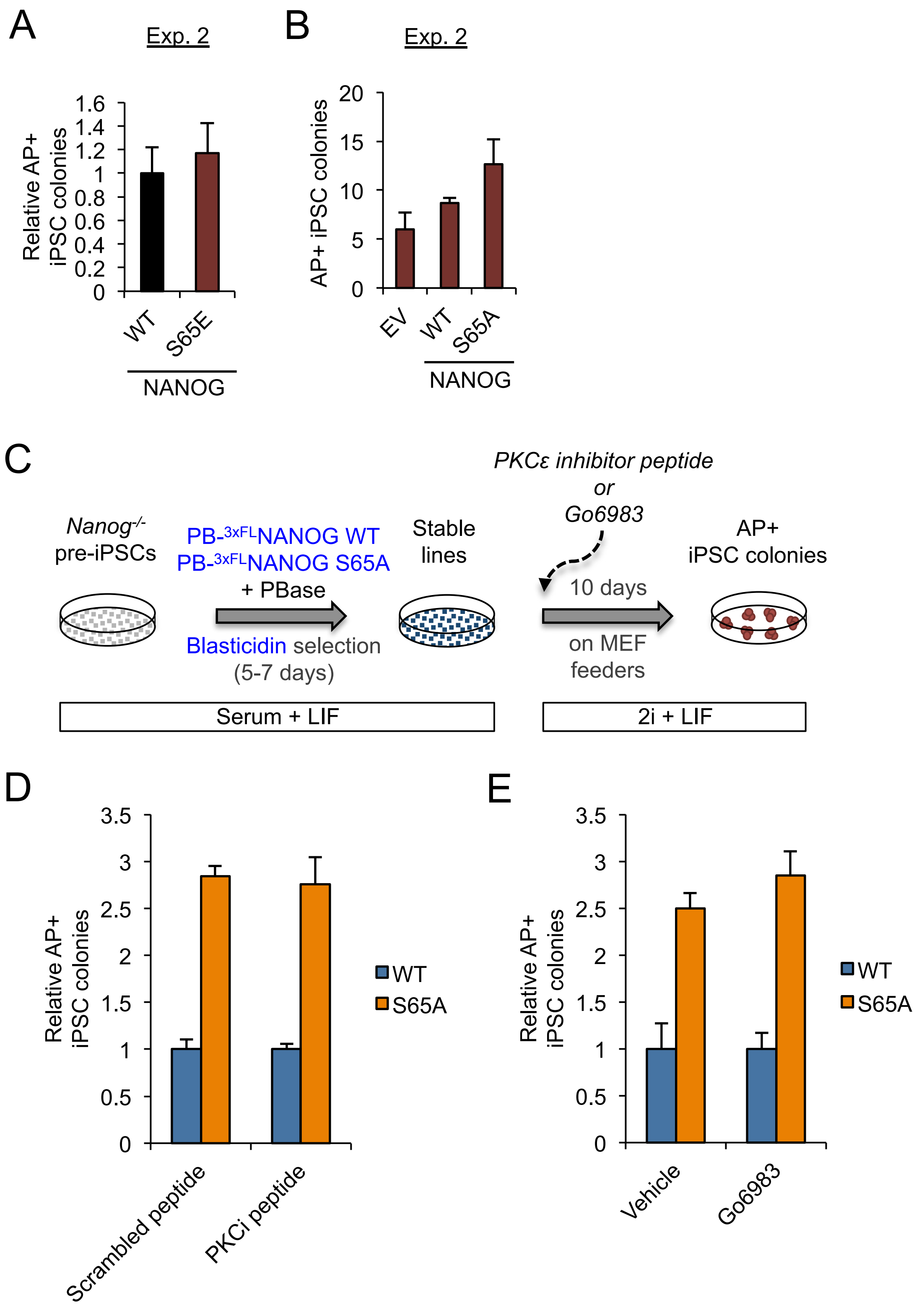
A
Exp. 2
Relative AP+ iPSC colonies
1.6
1.4
1.2
1
0.8
0.6
0.4
0.2
0
WT
S65E
NANOG
B
Exp. 2
AP+ iPSC colonies
20
15
10
5
0
EV
WT
S65A
NANOG
C
Nanog-/- pre-iPSCs
PB-3xFLNANOG WT
PB-3xFLNANOG S65A
+ PBase
Blasticidin selection (5-7 days)
Stable lines
PKCε inhibitor peptide or Go6983
10 days
on MEF feeders
AP+ iPSC colonies
Serum + LIF
2i + LIF
D
Relative AP+ iPSC colonies
3.5
3
2.5
2
1.5
1
0.5
0
Scrambled peptide
PKCi peptide
WT
S65A
E
Relative AP+ iPSC colonies
3.5
3
2.5
2
1.5
1
0.5
0
Vehicle
Go6983
WT
S65A

Figure S1

**Figure S2 (related to Figure 4). Pluripotency factors are preferentially associated with NANOG S65A pre-iPSCs**

(A) Western blot quantification for cytoplasmic (C), soluble nuclear (N), and chromatin-bound nuclear (B) protein lysates in $^{3xFLAG}$NANOG WT and $^{3xFLAG}$NANOG S65A pre-iPSCs. Percentages for C, N, and B fractions are based on FLAG/GAPDH, FLAG/HDAC1, and FLAG/H3 western blot intensities, respectively.

(B) SILAC IP-MS peaks showing increased abundance of REX1 and DAX1 in NANOG S65A pre-iPSCs, compared to NANOG WT pre-iPSCs.

(C) *Nanog* enhancer-driven luciferase assay to compare intrinsic transactivation activities of NANOG WT and S65A in HEK 293T cells using three different concentrations of DNA. Data are presented as average ± SD (n = 3 technical replicates per DNA concentration).

(D) Structural prediction and modeling of NANOG WT and NANOG S65A. The S65 residue in NANOG WT is highlighted yellow, whereas the S65A mutation in NANOG S65A is highlighted in red. Pink ribbons represent α-helices. Structural predictions were generated using the I-TASSER online platform, and modelling was performed using Jmol viewer.

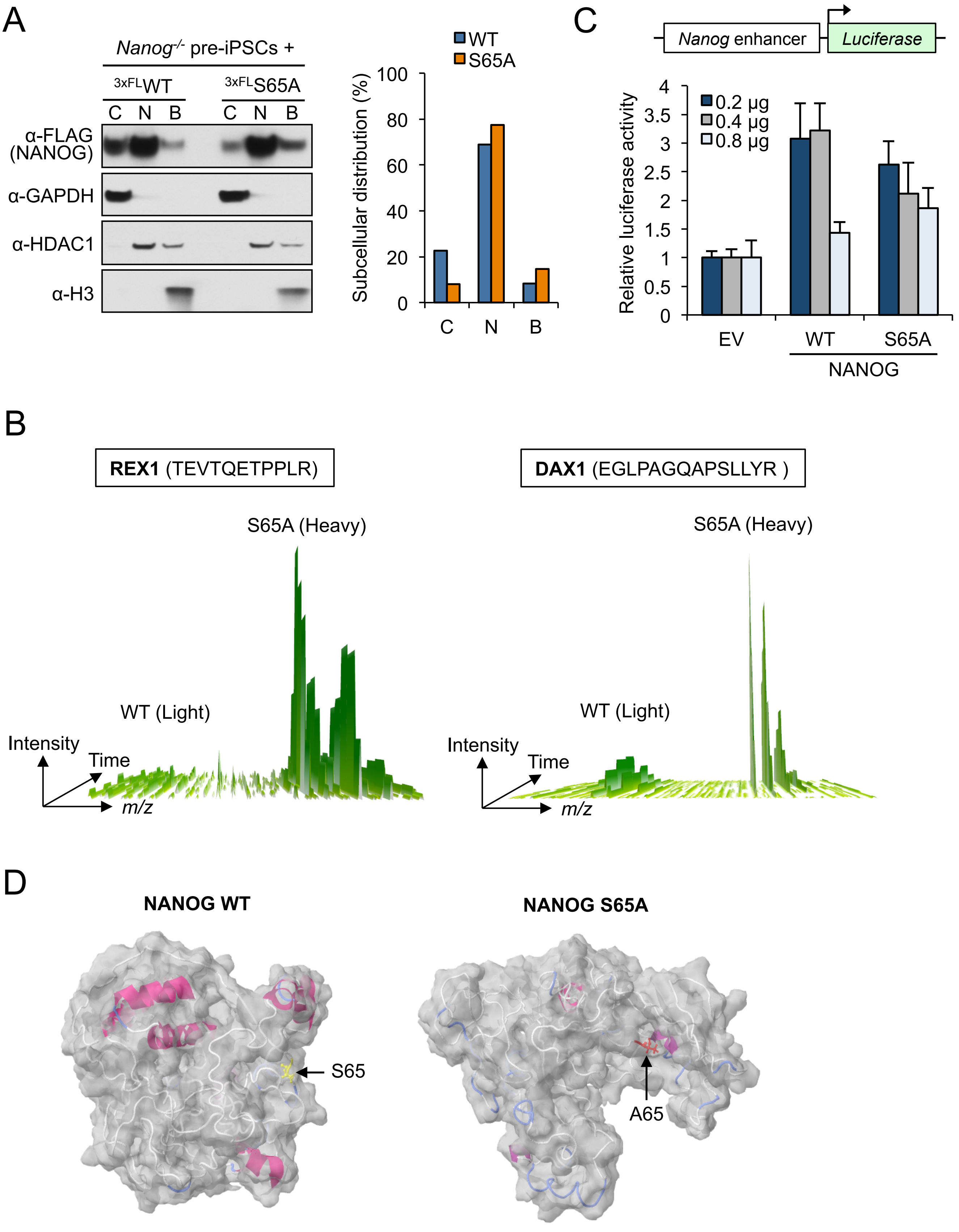
A
Nanog-/- pre-iPSCs +
3xFLWT
3xFLS65A
C N B
C N B
α-FLAG (NANOG)
α-GAPDH
α-HDAC1
α-H3
WT
S65A
Subcellular distribution (%)
100
80
60
40
20
0
C
N
B
C
Nanog enhancer
Luciferase
Relative luciferase activity
4
3.5
3
2.5
2
1.5
1
0.5
0
0.2 μg
0.4 μg
0.8 μg
EV
WT
S65A
NANOG
B
REX1 (TEVTQETPPLR)
DAX1 (EGLPAGQAPSLLYR )
S65A (Heavy)
WT (Light)
Intensity
Time
m/z
S65A (Heavy)
WT (Light)
Intensity
Time
m/z
D
NANOG WT
NANOG S65A
S65
A65

Figure S2

## SUPPLEMENTAL TABLES

**Table S1. List of qRT-PCR primers used in this study:**

| Gene | Forward | Reverse |
|---|---|---|
| *Esrrb* | CAGGCAAGGATGACAGACG | GAGACAGCACGAAGGACTGC |
| *Oct4 (Endo.)* | TCTTTCCACCAGGCCCCCGGCTC | TGCGGGCGGACATGGGGAGATCC |
| *Sall4* | AATGCTGTGCCGAGTTCTTT | GTGCCCAGCTTCTTCAAGTC |
| *Dax1* | ATCTGGAAGCAGGGCAAGTA | TCCTGTACCGCAGCTATGTG |
| *Tet1* | ATTGAGGTGGAGAAGTGGG | GGAGAAGGGTTGGTTTGC |
| *β-actin* | CACAGCTTCTTTGCAGCTCCTT | CGTCATCCATGGCGAACTG |

## SUPPLEMENTAL EXPERIMENTAL PROCEDURES

### ESC culture and LIF withdrawal colony formation assays

*Nanog* conditional knockout (NgcKO) mESCs were generated as described (Das et al., 2011) and were maintained on gelatin-coated cell culture dishes in standard mESC ("serum + LIF") culture medium [DMEM high glucose (Gibco), 15% heat inactivated FBS (Corning), Pen/Strep (Gibco), Nucleoside mix (Sigma), L-glutamine (Gibco), MEM Non-essential amino acids (Gibco), β-mercaptoethanol (0.1 mM), and recombinant LIF (20 ng/mL)]. ESCs were stably transfected with PB transposon vectors along with PB transposase (PBase) in a 1:2 PB:PBase ratio. Transfections for pre-iPSCs were performed in suspension using 1 µL of Lipofectamine 2000 transfection reagent (Invitrogen) per µg of transfected DNA. 24 hours after transfection, stable line selection began with 10-20 µg/mL Blasticidin S for 5-7 days, with passaging of cells at least once during drug selection.

For LIF withdrawal colony formation assays, NgcKO ESCs were seeded in serum + LIF at a density of 1000 cells per well of a 6-well gelatin-coated plate and allowed to attach for 24 hours. The next day, the culture medium was switched to standard mESC medium without LIF plus 1 µg/mL of doxycycline (Dox). This "Serum – LIF + Dox" medium was changed daily with fresh Dox. On day 5 or 6, plates were stained for alkaline phosphatase (AP) activity (Sigma) and colonies were counted under brightfield microscopy.

### IP-MS identification of endogenous NANOG phosphorylation sites

J1 mouse ESCs were expanded to 4 15-cm gelatin-coated cell culture dishes in standard serum + LIF medium supplemented with 1x PhosSTOP phosphatase inhibitor cocktail (Roche, 04906845001). Cells were trypsinized, pelleted, and washed briefly with cold PBS. Pellets were resuspended twice in cold Nuclear Extract Buffer A [10 mM HEPES (pH 8.0), 1.5 mM $MgCl_2$, 10 mM KCl] supplemented with 0.2 mM PMSF, 1x protease inhibitor cocktail (Sigma, P8340), and 1x PhosSTOP to eliminate cytoplasmic lysates. The remaining nuclear pellets were then resuspended in Lysis Buffer (same as above for western blotting) to extract nuclear lysates, and cell membranes and debris were removed by centrifugation for 15 min at 21,000 x g at 4 °C. The salt concentration of nuclear extracts was then increased to 500 mM NaCl to remove any contaminating NANOG interacting partners.

One hundred and fifty microliter (µL) of Protein G agarose (Roche, 11243233001) beads were equilibrated in BSA Buffer [1% BSA in PBS] and then rotated overnight at 4 °C with 40 µg of α-NANOG antibody (Bethyl, A300-397A-2). The next day, nuclear extracts were added to the bead/antibody mixture and rotated for 3 hours at 4 °C. Beads were then washed 4 times in Wash Buffer [250 mM NaCl, 20 mM Tris (pH 7.6), 20% glycerol, 0.05% NP-40, 0.2 mM EDTA, 0.2 mM PMSF], and protein was eluted twice from the beads by adding 4x SDS-DTT elution buffer and boiling at 95 °C for 5 minutes each time. Eluted protein was then concentrated using Amicon Ultra 3K centrifugal filters (Millipore) and subjected to SDS-PAGE. Finally, whole lanes were excised from the gel and subjected to LC-MS/MS mass spectrometry analysis.

Samples were reconstituted in 5-10 µL of HPLC solvent A (2.5% acetonitrile, 0.1% formic acid). A nano-scale reverse-phase HPLC capillary column was created by packing 5 µm C18 spherical silica beads into a fused silica capillary (100 µm inner diameter x 12 cm length) with a flame-draw tip. After equilibrating the column, each sample was loaded onto the column. A gradient of acetonitrile from 2.5% to 97.5% was used to elute the peptides. As peptides eluted, they were subjected to electrospray ionization and then entered into an LTQ-Orbitrap-Velos mass spectrometer (Thermo Finnigan) with collision-induced dissociation (CID). Eluting peptide were detected, isolated, and fragmented to produce a tandem mass spectrum of specific fragment ions for each peptide. MS data were processed by Thermo Proteome Discoverer software with SEQUEST engine against Swiss-Prot mouse protein sequence database. All NANOG phosphorylation sites were confirmed by manual annotation of the MS/MS spectrum. Information of the fragment ions refers to the MS-product utility at UCSF Proteinprospector (http://prospector.ucsf.edu).

### Pre-iPSC reprogramming

*Nanog*$^{-/-}$ neural stem cell (NSC)-derived pre-iPSCs were generated and used for reprogramming as described (Silva et al., 2009). Briefly, pre-iPSCs maintained in serum + LIF culture medium on gelatinized cell culture dishes, and were stably transfected with combinations of PiggyBac-CAG (PB) transposon vectors along with PB transposase (PBase) in a 1:2 PB:PBase ratio. Transfections and Blasticidin selection of pre-iPSCs were performed exactly as described above for NgcKO ESCs.

For pre-iPSC reprogramming, 1.0 x $10^4$ pre-iPSCs were seeded after selection onto gelatin-coated 12-well plates on top of a feeder layer of irradiated mouse embryonic fibroblasts (MEFs) (1.0 x $10^6$ MEFs per 12-well plate) and grown in serum + LIF for 2 days before medium switch to serum-free, N2B27 medium [DMEM/F12 (Gibco) and Neurobasal (Gibco) media were used in a 1:1 ratio, N2 supplement (Gibco), B27 supplement (Gibco), L-glutamine (Gibco), Pen/Strep (Gibco), and β-mercaptoethanol (0.1 mM)] supplemented with recombinant LIF (20 ng/mL), MEK inhibitor (PD325901; 1 μM final), and GSK3β inhibitor (CHIR99021; 3 μM final) ("2i + LIF" medium). Beginning on day 6 of reprogramming, 200 μg/mL G418 was added to 2i + LIF medium to positively select for activation of endogenous *Nanog* elements (Chambers et al., 2007). On day 10 in 2i + LIF, plates were stained for AP activity and iPSC colonies were counted under brightfield microscopy.

Protein kinase C epsilon (PKCε) inhibition during pre-iPSCs reprogramming was performed using 10 μM PKCε peptide inhibitor (Santa Cruz, sc-3095) or 10 μM scrambled peptide negative control (Santa Cruz, sc-3100). Pan-PKC inhibition was performed using 5 μM Go6983 (Tocris, 2285) or DMSO vehicle control. All inhibitors were added on day 0 of reprogramming in 2i + LIF.

**MEF reprogramming**

MEF reprogramming was performed as described (Vidal et al., 2014) with some modifications. Briefly, 3.0 x $10^4$ reprogrammable MEFs containing a Dox-inducible OKSM cassette were infected with retroviral NANOG WT, NANOG S65A, or EV control. The next day, 2000 infected MEFs/well were seeded on top of a feeder layer of irradiated mouse embryonic fibroblast feeders on a 6-well plate coated with gelatin, in "Dox + 3c"-containing ESC medium. On day 6, medium was switched to ESC medium without Dox or 3c, and plates were stained for alkaline phosphatase activity on day 10.

**SILAC IP-MS to compare NANOG WT and S65A interactomes in pre-iPSCs**

SILAC IP-MS was performed as described (Ding et al., 2015) with some modifications. Briefly, *Nanog*$^{-/-}$ pre-iPSCs expressing $^{3xFLAG}$NANOG WT or $^{3xFLAG}$NANOG S65A were each expanded to 8 15-cm dishes after culturing for 2 weeks in SILAC ESC medium [SILAC DMEM (Thermo Scientific, 89985), 10% dialyzed SILAC FBS (Thermo Scientific, 88440), Pen/Strep (Gibco), Nucleoside mix (Sigma), L-glutamine (Gibco), MEM Non-essential amino acids (Gibco), β-mercaptoethanol (0.1 mM), and recombinant LIF (20 ng/mL)] supplemented with either light or heavy lysine and arginine. Pre-iPSC nuclear extracts containing either $^{3xFLAG}$NANOG WT or $^{3xFLAG}$NANOG S65A were pre-cleared with 100 μL of Protein G agarose beads (Roche, cat.# 11243233001) rotating overnight at 4 °C. The next day, $^{3xFLAG}$NANOG WT or $^{3xFLAG}$NANOG S65A were immunoprecipitated using 200 μL of EZview red α-FLAG M2 affinity gel beads (Sigma), and were rotated at 4 °C for 3 hours. After washing beads, $^{3xFLAG}$NANOG WT or $^{3xFLAG}$NANOG S65A were eluted from α-FLAG beads with a 3xFLAG peptide (Sigma, F4799) solution (0.5 mg/mL). Eluted protein was then concentrated using Amicon Ultra 3K centrifugal filters (Millipore), quantified, mixed in a 1:1 ratio for each sample, and then subjected to SDS-PAGE. Finally, whole lanes were excised from the gel and subjected to quantitative LC-MS/MS mass spectrometry analysis.

LC-MS/MS analysis was performed as described earlier for NANOG phospho site mapping. MS data were processed by MaxQuant software with Andromeda search engine (http://www.maxquant.org). Spectrum was search against the Swiss-Prot mouse protein sequence database. All parameters followed the default setting. A cutoff of 2-fold change was applied for the normalized H/L ratios for the quantified proteins.

**Structural prediction and modeling of NANOG WT and S65A**

The full-length amino acid sequences for NANOG WT or NANOG S65A were submitted in FASTA format to the I-TASSER online platform for structural prediction (http://zhanglab.ccmb.med.umich.edu/I-TASSER/). For each submission, I-TASSER outputted the top 5 structural prediction models in PDB file format, which were quantitatively scored on confidence (C-score) based on threading template alignments and structural assembly simulations. Predicted structures for NANOG WT and NANOG S65A with the highest C-scores (indicative of highest confidence) were selected from their respective top 5 models. PDB files for NANOG WT and NANOG S65A were then manipulated in Jmol Viewer to generate the models presented.

**Western blotting**

Whole cell lysates from stable NgcKO ESCs and *Nanog*$^{-/-}$ pre-iPSCs were harvested using Lysis Buffer [50 mM HEPES (pH 7.6), 250 mM NaCl, 0.1% NP-40, 0.2 mM EDTA, 1.4 mM β-mercaptoethanol, 0.2 mM PMSF, 1x

protease inhibitor cocktail (Sigma, P8340)]. Lysates were subjected to SDS-PAGE using Mini-PROTEAN precast TGX 4-20% polyacrylamide gels (Bio-Rad). After transfer, membranes were blocked with 5% milk in TBS + 0.1% Tween, and then blotted with the following primary antibodies: α-FLAG M2 (Sigma, F3165), α-GAPDH (ProteinTech, 10494-1-AP), α-NANOG (Bethyl, A300-397A-2), α-VINCULIN (Abcam, ab129002), α-HDAC1 (Bethyl, A300-713A), α-HISTONE 3 (H3) (Abcam, ab1791), α-DAX1 (Santa Cruz, sc-841, K-17), α-OCT4 (Santa Cruz, sc-5279, C-10), α-SALL4 (Santa Cruz, sc-101147, EE-30), α-TET1 (Millipore, 09-872), α-ESRRB (Abcam, ab12986), and the following secondary antibodies: donkey α-mouse IgG-HRP (Santa Cruz, sc-2096), goat α-rabbit IgG-HRP (Santa Cruz, sc-2004). Western blot bands were quantified using ImageJ64 software by determining the FLAG/VINCULIN signal ratio for each sample. These ratios were then normalized to NANOG WT levels, and were set to 1.

**Multiple sequence alignment of mammalian NANOG proteins**
FASTA sequences for mouse (*Mus musculus*), human (*Homo sapiens*), chimpanzee (*Pan troglodytes*), bovine (*Bos taurus*), rat (*Rattus norvegicus*), macaque (*Macaca fascicularis*), and pig (*Sus scrofa*) NANOG N-terminal domains were obtained from UniProt (www.uniprot.org) and were aligned using the Clustal Omega online tool (www.ebi.ac.uk/Tools/msa/clustalo/).

**Statistical analysis**
Statistical analyses for all reprogramming experiments and ESC colony formation assays were performed using an unpaired, two-tailed Student's t-test, with significance values indicated in the figure legends. All error bars throughout the figures represent standard deviation (SD).

**Protein stability measurement**
Pre-iPSCs expressing $^{3xFLAG}$NANOG WT or $^{3xFLAG}$NANOG S65A were treated with 50 μg/mL of cycloheximide (Sigma, C4859) over a 6-hour time course. Whole cell lysates were extracted and subjected to western blotting as described above. Western blot bands were also quantified as described above, and were then plotted normalized to untreated (0 hours) samples, set to 100%. Protein half-lives of NANOG WT and NANOG S65A were calculated using linear regression analysis in Microsoft Excel to determine the time at which 50% of each protein was remaining.

**Immunofluorescence**
Immunofluorescence on pre-iPSCs expressing $^{3xFLAG}$NANOG WT or $^{3xFLAG}$NANOG S65A was performed as described (Gingold et al., 2014). Briefly, pre-iPSCs were cultured on glass slides, fixed in 4% paraformaldehyde, washed with PBS, and then permeabilized with 0.25% Triton X-100. Next, fixed cells were blocked with 10% BSA in PBS and then incubated overnight at 4 °C with the following primary antibodies: α-FLAG M2 (Sigma, F3165), α-GAPDH (ProteinTech, 10494-1-AP), and the following secondary antibodies: donkey anti-mouse AlexaFluor 488, donkey anti-rabbit RRX. DAPI was mixed with secondary antibodies as a nuclear marker. Fixed cells were then washed 3x in PBS and mounted for imaging by fluorescence microscopy.

**Luciferase assays**
*Nanog* enhancer-driven luciferase assays were performed as described (Ding et al., 2015) using 0.2 μg, 0.4 μg, and 0.8 μg of PB-empty vector (EV), NANOG WT, or NANOG S65A plasmids in HEK 293T cells.

**qRT-PCR**
Total RNA was extracted using the RNeasy kit (Qiagen) and cDNA was generated using qScript (Quanta, cat. # 95048). Relative expression levels were determined using the LightCycler 480 SYBR Green I Master mix (Roche, cat. # 4729749001), and expression levels were normalized to β-actin. qRT-PCR experiments were performed in triplicate on a LightCycler Real Time PCR System (Roche). See Table S1 for a list of all primers used in this study.

## SUPPLEMENTAL REFERENCES

Chambers, I., Silva, J., Colby, D., Nichols, J., Nijmeijer, B., Robertson, M., Vrana, J., Jones, K., Grotewold, L., Smith, A., 2007. Nanog safeguards pluripotency and mediates germline development. Nature 450, 1230–1234. doi:10.1038/nature06403

Gingold, J.A., Fidalgo, M., Guallar, D., Lau, Z., Sun, Z., Zhou, H., Faiola, F., Huang, X., Lee, D.-F., Waghray, A., Schaniel, C., Felsenfeld, D.P., Lemischka, I.R., Wang, J., 2014. A genome-wide RNAi screen identifies opposing functions of Snai1 and Snai2 on the Nanog dependency in reprogramming. Molecular Cell 56, 140–152. doi:10.1016/j.molcel.2014.08.014
