# Supplementary material for: Context-Dependent Functions of NANOG Phosphorylation in Pluripotency and Reprogramming
Source: Stem Cell Reports. 2017 Apr 27;8(5):1115–23. doi: 10.1016/j.stemcr.2017.03.023 (PMC5425684; doi:10.1016/j.stemcr.2017.03.023)
Supplement: Document S2. Article plus Supplemental Information [file mmc2.pdf]

Stem Cell Reports
Report
OPEN ACCESS

Context-Dependent Functions of NANOG Phosphorylation in Pluripotency and Reprogramming

Arven Saunders,[1,2,3,5] Dan Li,[1,2,3,5] Francesco Faiola,[1,3] Xin Huang,[1,3] Miguel Fidalgo,[1,3] Diana Guallar,[1,3] Junjun Ding,[1,3] Fan Yang,[1,3] Yang Xu,[4] Hongwei Zhou,[1,3] and Jianlong Wang[1,2,3,*]
[1]The Black Family Stem Cell Institute
[2]The Graduate School of Biomedical Sciences
[3]Department of Cell, Developmental, and Regenerative Biology
Icahn School of Medicine at Mount Sinai, New York, NY 10029, USA
[4]Division of Biological Sciences, University of California, San Diego, La Jolla, CA 92093, USA
[5]Co-first author
*Correspondence: jianlong.wang@mssm.edu
http://dx.doi.org/10.1016/j.stemcr.2017.03.023

SUMMARY

The core pluripotency transcription factor NANOG is critical for embryonic stem cell (ESC) self-renewal and somatic cell reprogramming. Although NANOG is phosphorylated at multiple residues, the role of NANOG phosphorylation in ESC self-renewal is incompletely understood, and no information exists regarding its functions during reprogramming. Here we report our findings that NANOG phosphorylation is beneficial, although nonessential, for ESC self-renewal, and that loss of phosphorylation enhances NANOG activity in reprogramming. Mutation of serine 65 in NANOG to alanine (S65A) alone has the most significant impact on increasing NANOG reprogramming capacity. Mechanistically, we find that pluripotency regulators (ESRRB, OCT4, SALL4, DAX1, and TET1) are transcriptionally primed and preferentially associated with NANOG S65A at the protein level due to presumed structural alterations in the N-terminal domain of NANOG. These results demonstrate that a single phosphorylation site serves as a critical interface for controlling context-dependent NANOG functions in pluripotency and reprogramming.

INTRODUCTION

The phospho-proteome in pluripotent stem cells has been extensively and systematically studied (Van Hoof et al., 2012), and has uncovered phosphorylated residues on pluripotency factors that play important roles in establishing and maintaining pluripotency. Despite the fact that NANOG was speculated to be a phospho-protein over a decade ago (Yates and Chambers, 2005), very little information is available regarding the status and functional implications of NANOG phosphorylation. Studies coupling immunoprecipitation with mass spectrometry (IP-MS) have found that human NANOG is phosphorylated at 11 different sites by ERK2 and CDK1 in human embryonic stem cells (ESCs) (Brumbaugh et al., 2014), and that mouse NANOG is phosphorylated at four different sites (Li et al., 2011; Moretto-Zita et al., 2010) by ERK1 as well as unidentified kinases (Kim et al., 2014). The specific role of phosphorylation in regulating NANOG function, however, remains elusive.

One study suggested that phosphorylation is important for maintaining NANOG stability in ESCs (Moretto-Zita et al., 2010). This study relied on ectopic expression of NANOG in HEK293 cells for identification of phosphorylation sites by IP-MS, and tested the functions of these phosphorylation sites with phospho-dead or phospho-mimic mutants in the presence of endogenous NANOG in wild-type (WT) mouse ESCs (mESCs) (Moretto-Zita et al., 2010). In contrast, another study reported that phosphorylation of NANOG by ERK1 during differentiation of ESCs decreases NANOG stability through ubiquitination-mediated degradation (Kim et al., 2014). Here we systematically investigated the function of NANOG phosphorylation in two biological settings within a physiological context where NANOG function is critical and endogenous NANOG interference with phospho-dead and phospho-mimic mutants is minimized. Our findings therefore contribute important functional data to the phospho-proteome in pluripotent stem cells, and improve our understanding of the key pluripotency regulator NANOG in controlling ESC pluripotency and somatic cell reprogramming.

RESULTS

NANOG Is Phosphorylated at Ser56/57 and Ser65 in mESCs

We performed IP-MS of endogenous NANOG in J1 mESCs (Figure 1A), and identified S56/57 and S65 as phosphorylated residues in the N terminus of NANOG (Figure 1B). We were unable, however, to distinguish phosphorylation between adjacent residues S56 and S57, similar to what others reported (Moretto-Zita et al., 2010). Comparison of our NANOG IP-MS analysis with that of other groups (Li et al., 2011; Moretto-Zita et al., 2010) revealed S65 as the only mouse NANOG phosphorylation site consistently identified by all studies to date, suggesting potential

© 2017 The Author(s). This is an open access article under the CC BY license (http://creativecommons.org/licenses/by/4.0/).
CrossMark

A B

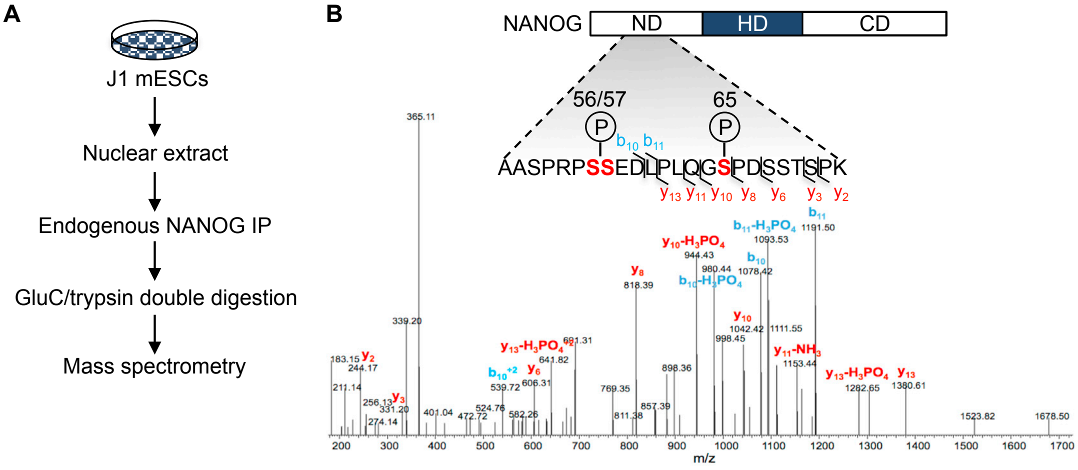

C

Reference	Phosphorylation site identified: S52	S56/57	S65	S77/78	Cell type	Mouse NANOG
This study		+	+		J1 mESCs	endogenous
Moretto-Zita *et al.*, 2010	+	+	+	+	HEK 293	ectopic
Li *et al.*, 2011			+		E14.1 mESCs	endogenous

D

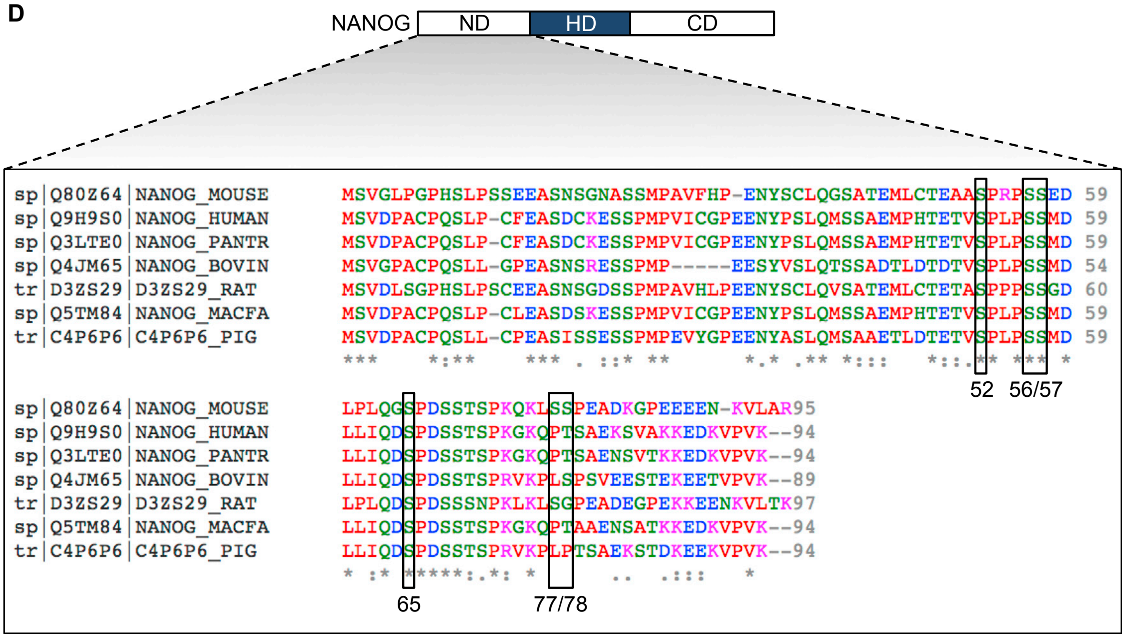

Figure 1. Identification of Phosphorylated Residues on Endogenous NANOG in ESCs

(A) Experimental design for endogenous NANOG IP-MS in J1 mESCs.

(B) Annotated spectrum identifying S56/57 and S65 as phosphorylated residues in the N terminus of NANOG. ND, N-terminal domain; HD, homeodomain; CD, C-terminal domain.

(C) Summary of all studies to date that have identified phosphorylated residues on mouse NANOG by IP-MS.

(D) Sequence alignment of the N-terminal domains of mouse, human, chimpanzee, bovine, rat, macaque, and pig NANOG (from top to bottom). Phospho-residues identified in mouse NANOG by all IP-MS studies are boxed. Asterisks indicate full amino acid conservation and colons indicate partial conservation. ND, N-terminal domain; HD, homeodomain; CD, C-terminal domain.

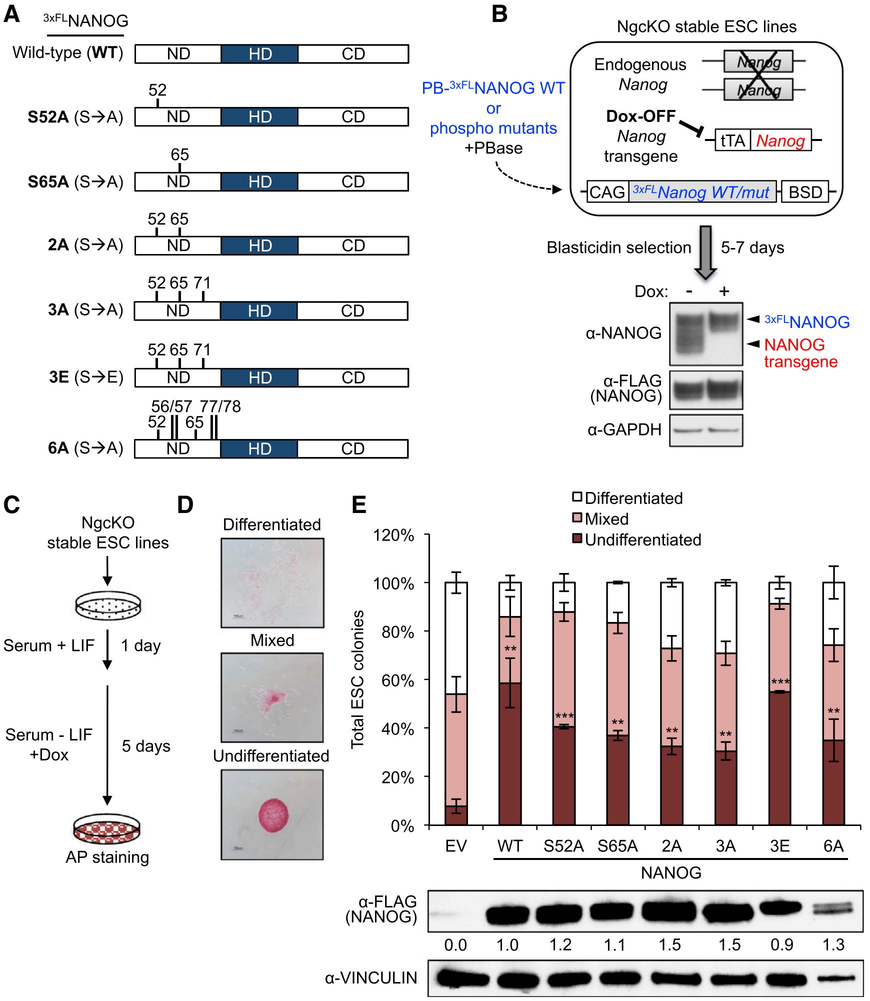

Figure 2. Phosphorylation Is Dispensable for NANOG to Sustain LIF-Independent mESC Self-Renewal

(A) 3xFLAG-tagged NANOG phospho-mutants used in this study. S→A denotes serine to alanine mutation and S→E serine-to-glutamic acid mutation of the indicated residues. ND, N-terminal domain; HD, homeodomain; CD, C-terminal domain.

(B) Schematic of the generation of *Nanog* conditional knockout (NgcKO) ESC lines stably expressing PiggyBac (PB) NANOG phospho-mutants. Western blot demonstrates the complete absence of the *Nanog* transgene after 24 hr of doxycycline (Dox; 1 μg/mL) treatment.

(C) Experimental design of the LIF withdrawal colony-formation assay.

(D) Representative images of ESC colony morphologies scored after AP staining.

(E) Phosphorylation is dispensable for NANOG to maintain ESC self-renewal. Data are presented as average percentages ± SD (n = 3 independent experiments; $^{**}p < 0.01$, $^{***}p < 0.001$). Statistical significances are relative to EV control. Western blots were performed on whole-cell lysates from stable ESC lines grown in serum + LIF conditions plus 24 hr of Dox (1 μg/mL) treatment.

importance for this residue in regulating NANOG function (Figure 1C). Interestingly, multiple sequence alignment of NANOG N-terminal domains revealed the full conservation of serines 52, 56/57, and 65 across several mammalian species (Figure 1D), suggesting that these phosphosites may be evolutionarily conserved to maintain NANOG function. In support of this hypothesis, human NANOG is also phosphorylated at S52, S56/57, and S65 in human ESCs (Brumbaugh et al., 2014), although the functions of these modifications remain undefined.

Phosphorylation Promotes NANOG Function in Sustaining mESC Self-Renewal

To comprehensively test the functions of all previously identified NANOG phosphorylation sites in the maintenance of pluripotency, we investigated how NANOG phospho-dead (S52A, S65A, 2A, 3A, and 6A) and phospho-mimic (3E) mutants (Figure 2A) could maintain pluripotency of ESCs in a *Nanog*$^{-/-}$ setting. We utilized doxycycline (Dox)-inducible *Nanog* conditional knockout (NgcKO) ESCs (Das et al., 2011) to generate stable cell lines overexpressing these NANOG phospho-mutants (Figure 2B) and tested the extent to which NANOG phospho-mutants could rescue leukemia inhibitory factor (LIF)-independent self-renewal in NgcKO ESCs.

We seeded NgcKO ESCs at clonal density, withdrew LIF, and added Dox for 5 days, then performed alkaline phosphatase (AP) staining to assess pluripotency status based on colony morphology (Figures 2C and 2D). As expected, cells expressing empty vector (EV) control generated only ~8% undifferentiated colonies out of the total number of colonies scored after 5 days of LIF withdrawal combined with Dox treatment (Figure 2E). We also noticed that phospho-dead mutants were less efficient than WT NANOG in rescuing ESC self-renewal upon LIF withdrawal. Notably, phospho-dead NANOG 3A was less efficient than its corresponding phospho-mimic NANOG 3E in forming undifferentiated colonies (Figure 2E), supporting that phosphorylation is beneficial to NANOG function in maintaining ESCs. Interestingly, however, we found

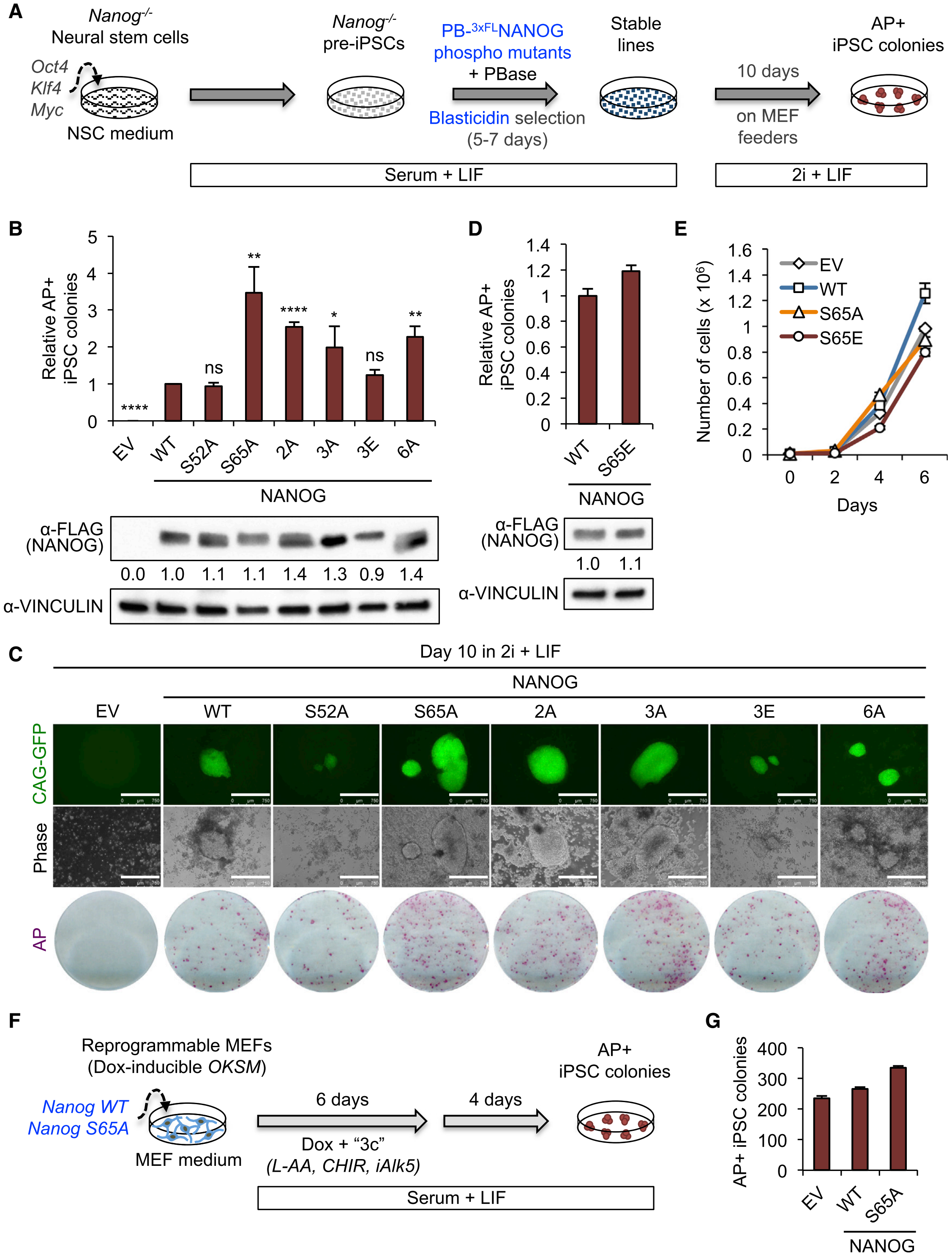

Figure 3. Loss of Phosphorylation Significantly Enhances NANOG Activity in Reprogramming

(A) The *Nanog*$^{-/-}$ neural stem cell (NSC)-derived pre-iPSC reprogramming system used for assessing NANOG phosphorylation gain or loss of function (see Experimental Procedures for more information).

(B) NANOG phospho-dead mutants are more efficient than NANOG WT in pre-iPSC reprogramming. Data are presented as average fold change of AP + iPSC colonies ± SD (n = 3 independent experiments; *p < 0.05, **p < 0.01, ****p < 0.0001; ns, not significant).

(legend continued on next page)

that all NANOG phospho-mutants could sustain LIF-independent self-renewal significantly better than EV control (Figure 2E), indicating that phosphorylation is not essential for NANOG to maintain self-renewal of ESCs.

Blocking Phosphorylation at Ser65 Enhances NANOG Reprogramming Activity

NANOG is critical for executing the final stage of reprogramming in various contexts (Costa et al., 2013; Silva et al., 2009); however, the role of NANOG phosphorylation in regulating this process has not been explored. We therefore tested how efficiently NANOG phospho-mutants could reprogram partially reprogrammed *Nanog*$^{-/-}$ somatic cells (pre-iPSCs) to naive induced pluripotent stem cells (iPSCs) (Figure 3A). As expected, we observed no iPSC colonies generated by pre-iPSCs expressing EV control after 10 days in serum-free, defined medium containing inhibitors of GSK3β and MEK/ERK (“2i + LIF”) (Figure 3B). We also did not observe any significant differences between the numbers of AP + iPSC colonies generated by NANOG WT and either NANOG S52A or NANOG 3E (a phospho-mimic). Surprisingly, however, we found that the NANOG phospho-dead mutants 2A, 3A, 6A, and especially S65A, generated significantly more AP + iPSC colonies than NANOG WT (Figures 3B and 3C). These data indicate that phosphorylation is not required for the ability of NANOG to execute the final stage of reprogramming, and importantly, that loss of phosphorylation significantly improves the capacity for NANOG to drive pre-iPSC reprogramming.

In particular, we found that out of all phospho-mutants tested, the single point mutant NANOG S65A was the most efficient in reprogramming *Nanog*$^{-/-}$ pre-iPSCs, reproducibly generating ~3.5-fold more AP + iPSC colonies than NANOG WT (Figures 3B and S1A). We next generated a phospho-mimic for this residue, NANOG S65E, and found no noticeable difference between the numbers of AP + iPSC colonies generated by either NANOG WT or NANOG S65E (Figures 3D and S1A), strongly suggesting that blocking phosphorylation at S65 can dramatically improve the reprogramming function of NANOG during the pre-iPSC to iPSC transition. Additionally we found that the proliferation rates of pre-iPSCs expressing EV, NANOG WT, NANOG S65A, and NANOG S65E were all comparable (Figure 3E), indicating that the increased activity of NANOG S65A in reprogramming was not due to an increase in proliferation rate.

To determine whether the same enhanced reprogramming activity of NANOG S65A could also be observed in conventional fibroblast reprogramming, we compared the ability of NANOG WT and S65A to enhance OKSM-mediated mouse embryonic fibroblast (MEF) reprogramming (Vidal et al., 2014) (Figure 3F). Addition of either NANOG WT or S65A consistently increased reprogramming efficiency over EV in two independent experiments, although NANOG S65A was appreciably more effective than NANOG WT in enhancing MEF reprogramming (Figures 3G and S1B).

Collectively, these results indicate that phosphorylation is dispensable for NANOG activity in reprogramming, and that blocking phosphorylation at S65 can enhance NANOG reprogramming activity.

S65A Mutation Does Not Affect NANOG Protein Stability or Subcellular Distribution

To determine whether the increased reprogramming activity of S65A over WT NANOG was due to enhanced protein stability, we treated NANOG WT and NANOG S65A pre-iPSCs with the translation inhibitor cycloheximide over a 6-hour time course. We did not observe any differences in the degradation rates or the protein half-lives of NANOG WT and NANOG S65A (Figures 4A and 4B), indicating that the enhanced reprogramming activity of NANOG S65A was not due to increased protein stability.

Next, we performed immunofluorescence for NANOG WT and NANOG S65A in pre-iPSCs and found that both were predominantly nuclear, with no obvious differences in overall distribution (Figure 4C), which we also confirmed by subcellular protein fractionation (Figure S2A).

Taken together, we conclude that the enhanced reprogramming activity of NANOG S65A is not due to increased protein stability or altered subcellular distribution.

Pluripotency Regulators Are Preferentially Associated with NANOG S65A in Pre-iPSCs

NANOG has been shown to prime the expression of certain pluripotency genes, such as *Esrrb* and *Oct4* in pre-iPSCs, to promote reprogramming efficiency (Costa et al., 2013). Using a candidate approach, we tested whether NANOG S65A

(C) Representative images of CAG-GFP + iPSC colonies as well as whole-well images of AP + iPSC colonies. Scale bars represent 750 μm.
(D) NANOG S65E phospho-mimic behaves like NANOG WT in pre-iPSC reprogramming. Data are presented as average fold change of AP + iPSC colonies ±SD (n = 3 technical replicates).
(E) Proliferation curve for *Nanog*$^{-/-}$ pre-iPSCs expressing EV, NANOG WT, NANOG S65A, and NANOG S65E.
(F) The doxycycline (Dox)-inducible MEF reprogramming system.
(G) NANOG S65A enhances OKSM-mediated MEF reprogramming. Data are presented as average fold change of AP + iPSC colonies ±SD (n = 3 technical replicates).
See also Figure S1.

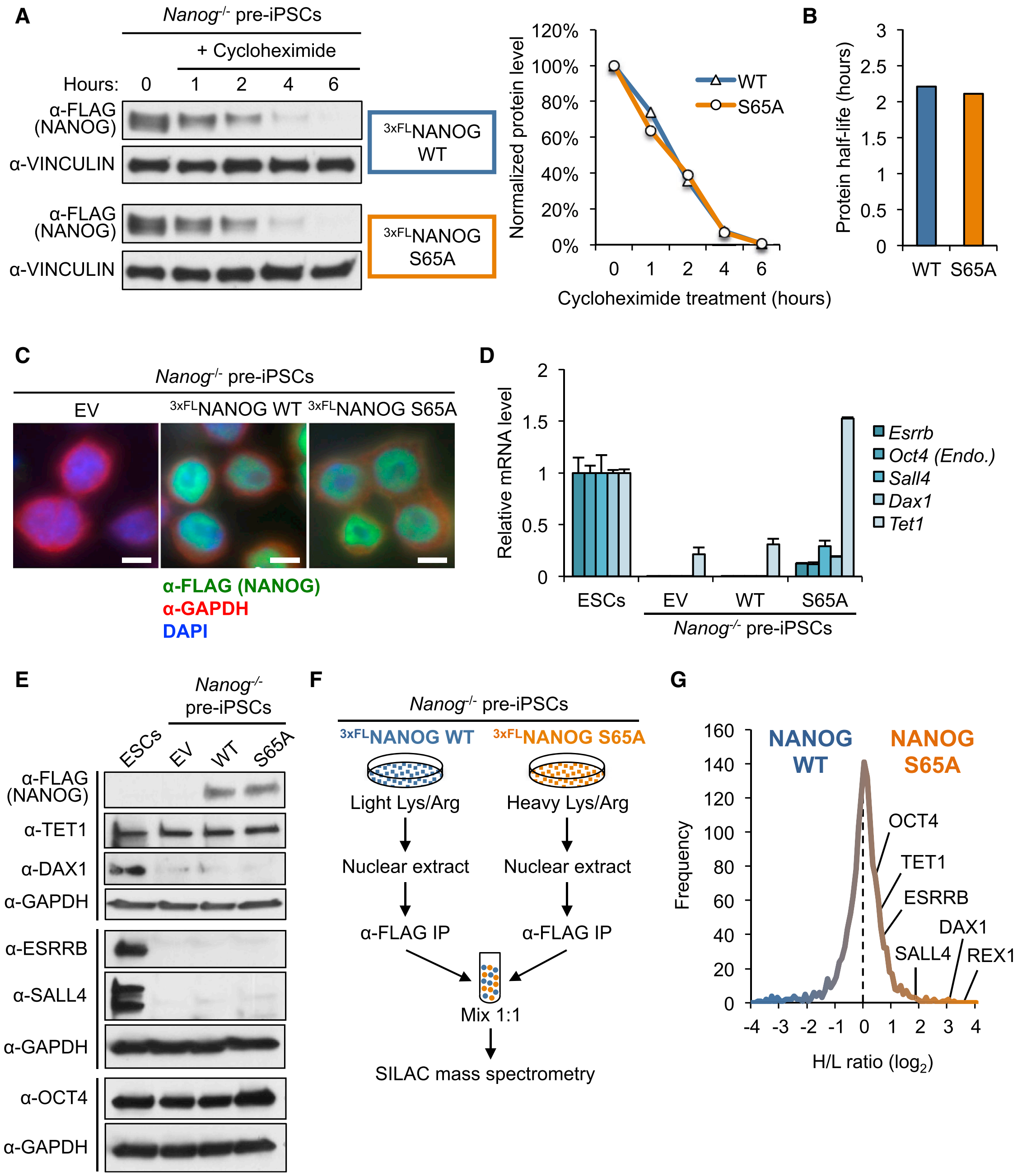

Figure 4. Pluripotency Factors Are Preferentially Associated with NANOG S65A Pre-iPSCs

(A) Western blot analysis of a 6-hr cycloheximide (50 μg/mL) time-course treatment in *Nanog*$^{-/-}$ pre-iPSCs expressing ^{3xFLAG}NANOG WT or ^{3xFLAG}NANOG S65A in serum + LIF culture conditions.

(B) Half-lives of NANOG WT and NANOG S65A were calculated using linear regression analysis of the data plotted in (A).

(C) Immunofluorescence for ^{3xFLAG}NANOG WT and S65A in pre-iPSCs. GAPDH and DAPI were used as cytoplasmic and nuclear markers, respectively. Scale bars represent 5 μm.

(D) RT-PCR analysis for *Esrrb*, endogenous (*Oct4* Endo.), *Sall4*, *Dax1*, and *Tet1* mRNA levels in ^{3xFLAG}NANOG WT and S65A pre-iPSCs, as well as WT E14T ESCs. RNA was collected from stable pre-iPSC lines immediately following blasticidin selection (see Figure 3A). Data are presented as average ± SD (n = 3 technical replicates), relative to *β-actin* housekeeping control, and each gene is normalized to the levels in E14T mESCs.

(legend continued on next page)

overexpression affected the expression levels of genes known to be critical for the reprogramming process. We found that the pluripotency-associated genes *Esrrb*, *Oct4*, *Sall4*, *Dax1*, and *Tet1* were all transcriptionally upregulated in NANOG S65A pre-iPSCs, compared with NANOG WT pre-iPSCs, although still far below their ESC levels (except for *Tet1*) (Figure 4D). Western blotting of whole-cell lysates revealed that, relative to their protein expression in ESCs, these pluripotency regulators were either non-detectable (e.g., DAX1, ESRRB, and SALL4) or equally abundant in NANOG WT and S65A pre-iPSCs when expressed at detectable levels (e.g., TET1 and OCT4) (Figure 4E), suggesting a potential post-transcriptional regulation of these pluripotency gene transcripts.

NANOG can physically interact and synergize with pluripotency regulators such as TET1 to promote pre-iPSC reprogramming (Costa et al., 2013). To overcome the western detection threshold for physical associations of NANOG WT and S65A with those transcriptionally activated pluripotency regulators in pre-iPSCs, we utilized a more sensitive approach, namely SILAC (stable isotope labeling by amino acids in cell culture)-based quantitative mass spectrometry, coupled with IP of NANOG to compare the WT and S65A NANOG interactomes in *Nanog*$^{-/-}$ pre-iPSCs (Figure 4F). As expected, the majority of proteins identified by SILAC IP-MS were equally abundant in both cell populations (heavy/light $\log_2$ ratio $\approx$ 0). Most importantly, however, we detected a preferential enrichment of all the pluripotency regulators tested in Figures 4E and 4F (TET1, DAX1, ESRRB, SALL4, and OCT4) in the NANOG S65A interactome compared with the NANOG WT interactome (Figures 4G and S2B).

Together, we conclude that loss of phosphorylation may endow NANOG S65A with increased affinity with other pluripotency-associated regulators, leading to enhanced reprogramming (see Discussion and Figure S2D).

DISCUSSION

Here we report our findings on the role of NANOG phosphorylation in the maintenance and establishment of pluripotency. While phosphorylation was not essential for NANOG to maintain pluripotency of ESCs, it seemed to be beneficial for NANOG to sustain ESC self-renewal. Conversely, however, we found that loss of phosphorylation promoted NANOG function in reprogramming.

What might be the underlying cause of such context-dependent functions of NANOG phosphorylation in pluripotency and reprogramming? We did not observe any differences in protein stability or subcellular localization between NANOG WT and S65A in *Nanog*$^{-/-}$ pre-iPSCs (Figure 4). The intrinsic transcriptional activity also does not seem to be affected in S65A mutant relative to WT NANOG, as we observed no noticeable difference in the abilities of WT and S65A NANOG to activate a *Nanog* enhancer-driven luciferase reporter (Figure S2C). Importantly, our highly sensitive SILAC IP-MS studies indicated that the NANOG S65A interactome in pre-iPSCs is preferentially enriched for the pluripotency factors ESRRB, OCT4, SALL4, DAX1, and TET1, compared with the WT NANOG interactome, in pre-iPSCs (Figure 4G). These factors often co-occupy ESC super-enhancers with NANOG, and have all been implicated in the reprogramming process (Costa et al., 2013; Huang and Wang, 2014). Therefore, it is highly likely that, despite minimal expression levels of DAX1, ESRRB, and SALL4, or equal abundance of TET1 and OCT4, in pre-iPSCs relative to ESCs (Figure 4E), these pluripotency factors may have a higher affinity with S65A than WT NANOG in forming active transcriptional regulatory complexes to mediate enhanced reprogramming.

Is there a structural implication for such preferential association of NANOG S65A with pluripotency regulators? By applying automated protein structure prediction and modeling for full-length NANOG WT and NANOG S65A using the I-TASSER platform (Roy et al., 2010), we observed an apparent unfolding of the N-terminal domain of S65A compared with WT NANOG (Figure S2D). The N-terminal domain has been shown to be dispensable for NANOG nuclear localization, and has been proposed to serve as an interface for interaction with co-factors important for transcriptional activities of NANOG in maintaining ESC self-renewal (Chang et al., 2009; Guo et al., 2009). Therefore, loss of phosphorylation may have endowed NANOG

(E) Western blot analyses of FLAG, TET1, DAX1, ESRRB, SALL4, OCT4, and GAPDH (loading control) protein levels in J1 mESCs, and in pre-iPSCs expressing EV, ^{3xFLAG}NANOG WT, and S65A pre-iPSC whole-cell lysates. Total lysates were collected from stable pre-iPSC lines immediately following blasticidin selection (see Figure 3A).
(F) Illustration of the SILAC mass spectrometry experiment performed on stable *Nanog*$^{-/-}$ pre-iPSCs expressing ^{3xFLAG}NANOG WT and S65A. ^{3xFLAG}NANOG WT pre-iPSCs were cultured in serum + LIF medium containing light isotope-labeled lysine and arginine, and ^{3xFLAG}NANOG S65A pre-iPSCs were cultured in serum + LIF medium containing heavy isotope-labeled lysine and arginine.
(G) Histogram of the frequency distribution of heavy/light (H/L) ratios ($\log_2$ scale) of all proteins identified by SILAC IP-MS. The pluripotency factors REX1, DAX1, SALL4, ESRRB, TET1, and OCT4 were identified as putative preferential interacting partners of ^{3xFLAG}NANOG S65A, compared with ^{3xFLAG}NANOG WT, in pre-iPSCs.
See also Figure S2.

S65A with an altered structure more conducive to association with those nuclear pluripotency regulators, leading to functional activation of the pluripotency program in reprogramming. Future studies applying X-ray crystallography to solve the full-length WT and S65A NANOG protein structures are warranted to confirm this hypothesis, which is currently a challenge in the field (Hayashi et al., 2015; Jauch et al., 2008). Alternatively, the preferential associations of S65A NANOG with these pluripotency regulators (Figure 4G) may also be due to their subtle increased protein levels that can only be detected by quantitative SILAC IP-MS we have employed. Of note, we found that the total OCT4 protein level in S65A pre-iPSCs was appreciably higher than that in WT pre-iPSCs (Figure 4E), likely resulting from endogenous *Oct4* reactivation.

Which kinase could be responsible for S65 phosphorylation? Mouse NANOG has so far only been found to be phosphorylated by ERK1 in differentiating mESCs at serine residues other than S65, resulting in reduced NANOG protein stability (Kim et al., 2014). Such reported destabilizing effects of ERK1-mediated phosphorylation at neighboring residues other than S65 is expected to be equally applicable to NANOG WT and S65A, and is consistent with increased NANOG protein levels in ESCs under 2i + LIF culture (Silva et al., 2009). While this may provide an explanation for the identical protein stability between NANOG WT and S65A in pre-iPSCs under serum + LIF culture (Figure 4A), it cannot explain the enhanced reprogramming activity of NANOG S65A over WT under standard 2i + LIF culture that contains a MEK/ERK inhibitor (Silva et al., 2008) (Figure 3). Interestingly, human NANOG has also been shown to be phosphorylated by protein kinase Cε (PKCε) in cancer cells (Bourguignon et al., 2009; Xie et al., 2013). However, we were unable to increase the reprogramming efficiency of NANOG WT to that of NANOG S65A by treating pre-iPSCs with either a PKCε-specific translocation inhibitor (Figures S1C and S1D) or a pan-PKC inhibitor Go6983 (Figures S1C and S1E), suggesting that PKC does not phosphorylate mouse NANOG. Future studies will be needed to identify the specific kinase responsible for S65 phosphorylation, and to address its impact on NANOG function in pluripotency and reprogramming.

EXPERIMENTAL PROCEDURES

NANOG Phosphorylation Mutants

NANOG phospho-mutant plasmids were generated as described by Moretto-Zita et al. (2010). These NANOG mutants were then subcloned via PCR into PiggyBac-CAG (PB) transposon vectors containing an N-terminal 3xFLAG epitope tag for stable overexpression. NANOG S65E phospho-mimic was generated via site-directed mutagenesis using the QuikChange Lightning Multi Site-Directed Mutagenesis Kit (Agilent Technologies). Following PCR amplification and subcloning, all phospho-mutant constructs were verified by sequencing.

Pre-iPSC and MEF Reprogramming

Nanog$^{-/-}$ neural stem cell (NSC)-derived pre-iPSCs were generated and used for reprogramming as described by Silva et al. (2009). In brief, 1.0×10^4 pre-iPSCs were seeded after selection onto gelatin-coated 12-well plates on top of irradiated MEFs and grown in serum + LIF for 2 days before medium switch to 2i + LIF medium (20 ng/mL LIF, 1 μM PD325901, and 3 μM CHIR99021). On day 10 in 2i + LIF, plates were stained for AP activity and iPSC colonies were counted under bright-field microscopy.

MEF reprogramming was performed as described by Vidal et al. (2014) with some modifications. In brief, 3.0×10^4 reprogrammable MEFs containing a Dox-inducible OKSM cassette were infected with retroviral NANOG WT, NANOG S65A, or EV control. The next day 34,000 infected MEFs/well were seeded on top of a feeder layer of irradiated MEF feeders on a 6-well plate coated with gelatin, in "Dox + 3c"-containing ESC medium. On day 6 the medium was switched to ESC medium without Dox or 3c, and plates were stained for AP activity on day 10.

SILAC IP-MS to Compare NANOG WT and S65A Interactomes in Pre-iPSCs

SILAC IP-MS was performed as described by Ding et al. (2015) with some modifications. In brief, *Nanog*$^{-/-}$ pre-iPSCs expressing ^{3xFLAG}NANOG WT or ^{3xFLAG}NANOG S65A were each expanded to 8 15-cm dishes after culturing for 2 weeks in SILAC ESC medium supplemented with either light or heavy lysine and arginine. Pre-iPSC nuclear extracts were pre-cleared with Protein G agarose beads rotating overnight at 4°C. The next day, ^{3xFLAG}NANOG WT or ^{3xFLAG}NANOG S65A were immunoprecipitated, washed, and eluted from α-FLAG beads with a 3xFLAG peptide solution. Eluted protein was then concentrated, quantified, mixed in a 1:1 ratio for each sample, and subjected to SDS-PAGE. Finally, whole lanes were excised from the gel and subjected to quantitative liquid chromatography-tandem mass spectrometry analysis.

SUPPLEMENTAL INFORMATION

Supplemental Information includes Supplemental Experimental Procedures, two figures, and one table and can be found with this article online at http://dx.doi.org/10.1016/j.stemcr.2017.03.023.

AUTHOR CONTRIBUTIONS

A.S. designed and performed experiments, analyzed data, and wrote the manuscript; D.L. performed experiments and analyzed data; F.F. and X.H. provided bioinformatics support and technical assistance; M.F., D.G., J.D., F.Y., Y.X., and H.Z. provided technical assistance, reagents, and helpful discussions; J.W. conceived the project, designed the experiments, analyzed data, and prepared and approved the manuscript.

ACKNOWLEDGMENTS

We thank Drs. D. Levasseur for the NgcKO ESCs, J. Silva for the *Nanog*$^{-/-}$ pre-iPSCs, and M. Stadtfeld for the reprogrammable

MEFs. This research was funded by grants from the NIH to J.W. (R01GM095942 and R21HD087722) and the Empire State Stem Cell Fund through New York State Department of Health (NYSTEM) to J.W. (C028103, C028121). J.W. is a recipient of an Irma T. Hirschl and Weill-Caulier Trusts Career Scientist Award. A.S. is an awardee of the Traineeship of NIDCR-Interdisciplinary Training in Systems and Developmental Biology and Birth Defects (T32HD075735).

Received: April 27, 2016
Revised: March 18, 2017
Accepted: March 28, 2017
Published: April 27, 2017

REFERENCES

Bourguignon, L.Y.W., Spevak, C.C., Wong, G., Xia, W., and Gilad, E. (2009). Hyaluronan-CD44 interaction with protein kinase C promotes oncogenic signaling by the stem cell marker Nanog and the production of MicroRNA-21, leading to down-regulation of the tumor suppressor protein PDCD4, anti-apoptosis, and chemotherapy resistance in breast tumor cells. J. Biol. Chem. *284*, 26533–26546.

Brumbaugh, J., Russell, J.D., Yu, P., Westphall, M.S., Coon, J.J., and Thomson, J.A. (2014). Stem cell reports. Stem Cell Rep. *2*, 18–25.

Chang, D.F., Tsai, S.C., Wang, X.C., Xia, P., Senadheera, D., and Lutzko, C. (2009). Molecular characterization of the human NANOG protein. Stem Cells *27*, 812–821.

Costa, Y., Ding, J., Theunissen, T.W., Faiola, F., Hore, T.A., Shliaha, P.V., Fidalgo, M., Saunders, A., Lawrence, M., Dietmann, S., et al. (2013). NANOG-dependent function of TET1 and TET2 in establishment of pluripotency. Nature *495*, 370–374.

Das, S., Jena, S., and Levasseur, D.N. (2011). Alternative splicing produces Nanog protein variants with different capacities for self-renewal and pluripotency in embryonic stem cells. J. Biol. Chem. *286*, 42690–42703.

Ding, J., Huang, X., Shao, N., Zhou, H., Lee, D.-F., Faiola, F., Fidalgo, M., Guallar, D., Saunders, A., Shliaha, P.V., et al. (2015). Tex10 coordinates epigenetic control of super-enhancer activity in pluripotency and reprogramming. Cell Stem Cell *16*, 653–668.

Guo, Y., Zhang, J., Ye, L., Chen, M., Yao, D., Pan, G., Zhang, J., and Pei, D. (2009). The N-terminal domain is a transcriptional activation domain required for Nanog to maintain ES cell self-renewal. Chin. Sci. Bull. *54*, 3271–3277.

Hayashi, Y., Caboni, L., Das, D., Yumoto, F., Clayton, T., Deller, M.C., Nguyen, P., Farr, C.L., Chiu, H.-J., Miller, M.D., et al. (2015). Structure-based discovery of NANOG variant with enhanced properties to promote self-renewal and reprogramming of pluripotent stem cells. Proc. Natl. Acad. Sci. USA *112*, 4666–4671.

Huang, X., and Wang, J. (2014). The extended pluripotency protein interactome and its links to reprogramming. Curr. Opin. Genet. Dev. *28*, 16–24.

Jauch, R., Ng, C.K.L., Saikatendu, K.S., Stevens, R.C., and Kolatkar, P.R. (2008). Crystal structure and DNA binding of the homeodomain of the stem cell transcription factor Nanog. J. Mol. Biol. *376*, 758–770.

Kim, S.-H., Kim, M.O., Cho, Y.-Y., Yao, K., Kim, D.J., Jeong, C.-H., Yu, D.H., Bae, K.B., Cho, E.J., Jung, S.K., et al. (2014). ERK1 phosphorylates Nanog to regulate protein stability and stem cell self-renewal. Stem Cell Res. *13*, 1–11.

Li, Q.R., Xing, X.B., Chen, T.T., Li, R.X., Dai, J., Sheng, Q.H., Xin, S.M., Zhu, L.L., Jin, Y., Pei, G., et al. (2011). Large scale phosphoproteome profiles comprehensive features of mouse embryonic stem cells. Mol. Cell Proteomics *10*, M110.001750.

Moretto-Zita, M., Jin, H., Shen, Z., Zhao, T., Briggs, S.P., and Xu, Y. (2010). Phosphorylation stabilizes Nanog by promoting its interaction with Pin1. Proc. Natl. Acad. Sci. USA *107*, 13312–13317.

Roy, A., Kucukural, A., and Zhang, Y. (2010). I-TASSER: a unified platform for automated protein structure and function prediction. Nat. Protoc. *5*, 725–738.

Silva, J., Barrandon, O., Nichols, J., and Kawaguchi, J. (2008). Promotion of reprogramming to ground state pluripotency by signal inhibition. PLos Biol. *6*, e253.

Silva, J., Nichols, J., Theunissen, T.W., Guo, G., van Oosten, A.L., Barrandon, O., Wray, J., Yamanaka, S., Chambers, I., and Smith, A. (2009). Nanog is the gateway to the pluripotent ground state. Cell *138*, 722–737.

Van Hoof, D., Krijgsveld, J., and Mummery, C. (2012). Proteomic analysis of stem cell differentiation and early development. Cold Spring Harb. Perspect. Biol. *4*, a008177. http://dx.doi.org/10.1101/cshperspect.a008177.

Vidal, S.E., Amlani, B., Chen, T., Tsirigos, A., and Stadtfeld, M. (2014). Combinatorial modulation of signaling pathways reveals cell-type-specific requirements for highly efficient and synchronous iPSC reprogramming. Stem Cell Rep. *3*, 574–584.

Xie, X., Piao, L., Cavey, G.S., Old, M., Teknos, T.N., Mapp, A.K., and Pan, Q. (2013). Phosphorylation of Nanog is essential to regulate Bmi1 and promote tumorigenesis. Oncogene *33*, 2040–2052.

Yates, A., and Chambers, I. (2005). The homeodomain protein Nanog and pluripotency in mouse embryonic stem cells. Biochem. Soc. Trans. *33*, 1518–1521.

Stem Cell Reports, Volume *8*

Supplemental Information

Context-Dependent Functions of NANOG Phosphorylation in Pluripotency and Reprogramming

Arven Saunders, Dan Li, Francesco Faiola, Xin Huang, Miguel Fidalgo, Diana Guallar, Junjun Ding, Fan Yang, Yang Xu, Hongwei Zhou, and Jianlong Wang

SUPPLEMENTAL FIGURES AND FIGURE LEGENDS

Figure S1 (related to Figure 3). Loss of phosphorylation enhances NANOG activity in reprogramming

(A) NANOG S65E phospho mimic behaves like NANOG WT in pre-iPSC reprogramming. Data from an independent experiment (biological replicate) are presented as average fold change of AP+ iPSC colonies ± SD (n = 3 technical replicates).

(B) NANOG S65A enhances OKSM-mediated MEF reprogramming. Data are presented as average fold change of AP+ iPSC colonies ± SD (n = 3 technical replicates).

(C) The *Nanog*$^{-/-}$ neural stem cell (NSC)-derived pre-iPSC reprogramming system used for assessing PKC inhibition.

(D) PKCε peptide inhibitor does not affect the enhanced reprogramming capacity of NANOG S65A over WT. Data are presented as average fold change of AP+ iPSC colonies ± SD (n = 3 technical replicates).

(E) Pan-PKC inhibition does not affect the enhanced reprogramming capacity of NANOG S65A over WT. Data are presented as average fold change of AP+ iPSC colonies ± SD (n = 3 technical replicates).

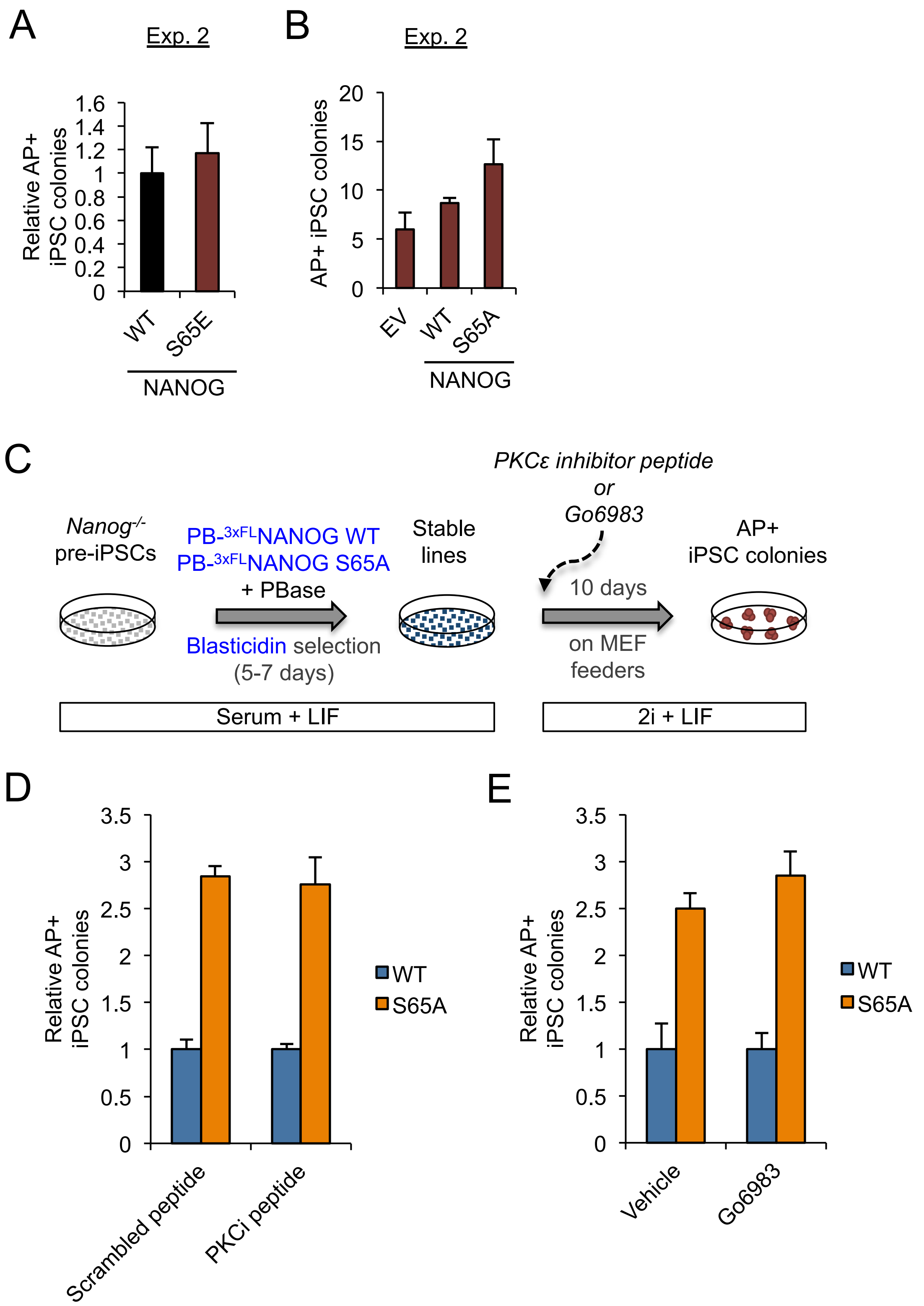
A
Exp. 2
Relative AP+ iPSC colonies
1.6
1.4
1.2
1
0.8
0.6
0.4
0.2
0
WT
S65E
NANOG
B
Exp. 2
AP+ iPSC colonies
20
15
10
5
0
EV
WT
S65A
NANOG
C
PKCε inhibitor peptide
or
Go6983
Nanog-/-
pre-iPSCs
PB-3xFLNANOG WT
PB-3xFLNANOG S65A
+ PBase
Blasticidin selection
(5-7 days)
Stable
lines
10 days
on MEF
feeders
AP+
iPSC colonies
Serum + LIF
2i + LIF
D
Relative AP+ iPSC colonies
3.5
3
2.5
2
1.5
1
0.5
0
WT
S65A
Scrambled peptide
PKCi peptide
E
Relative AP+ iPSC colonies
3.5
3
2.5
2
1.5
1
0.5
0
WT
S65A
Vehicle
Go6983

Figure S1

Figure S2 (related to Figure 4). Pluripotency factors are preferentially associated with NANOG S65A pre-iPSCs

(A) Western blot quantification for cytoplasmic (C), soluble nuclear (N), and chromatin-bound nuclear (B) protein lysates in ^{3xFLAG}NANOG WT and ^{3xFLAG}NANOG S65A pre-iPSCs. Percentages for C, N, and B fractions are based on FLAG/GAPDH, FLAG/HDAC1, and FLAG/H3 western blot intensities, respectively.

(B) SILAC IP-MS peaks showing increased abundance of REX1 and DAX1 in NANOG S65A pre-iPSCs, compared to NANOG WT pre-iPSCs.

(C) *Nanog* enhancer-driven luciferase assay to compare intrinsic transactivation activities of NANOG WT and S65A in HEK 293T cells using three different concentrations of DNA. Data are presented as average ± SD (n = 3 technical replicates per DNA concentration).

(D) Structural prediction and modeling of NANOG WT and NANOG S65A. The S65 residue in NANOG WT is highlighted yellow, whereas the S65A mutation in NANOG S65A is highlighted in red. Pink ribbons represent α-helices. Structural predictions were generated using the I-TASSER online platform, and modelling was performed using Jmol viewer.

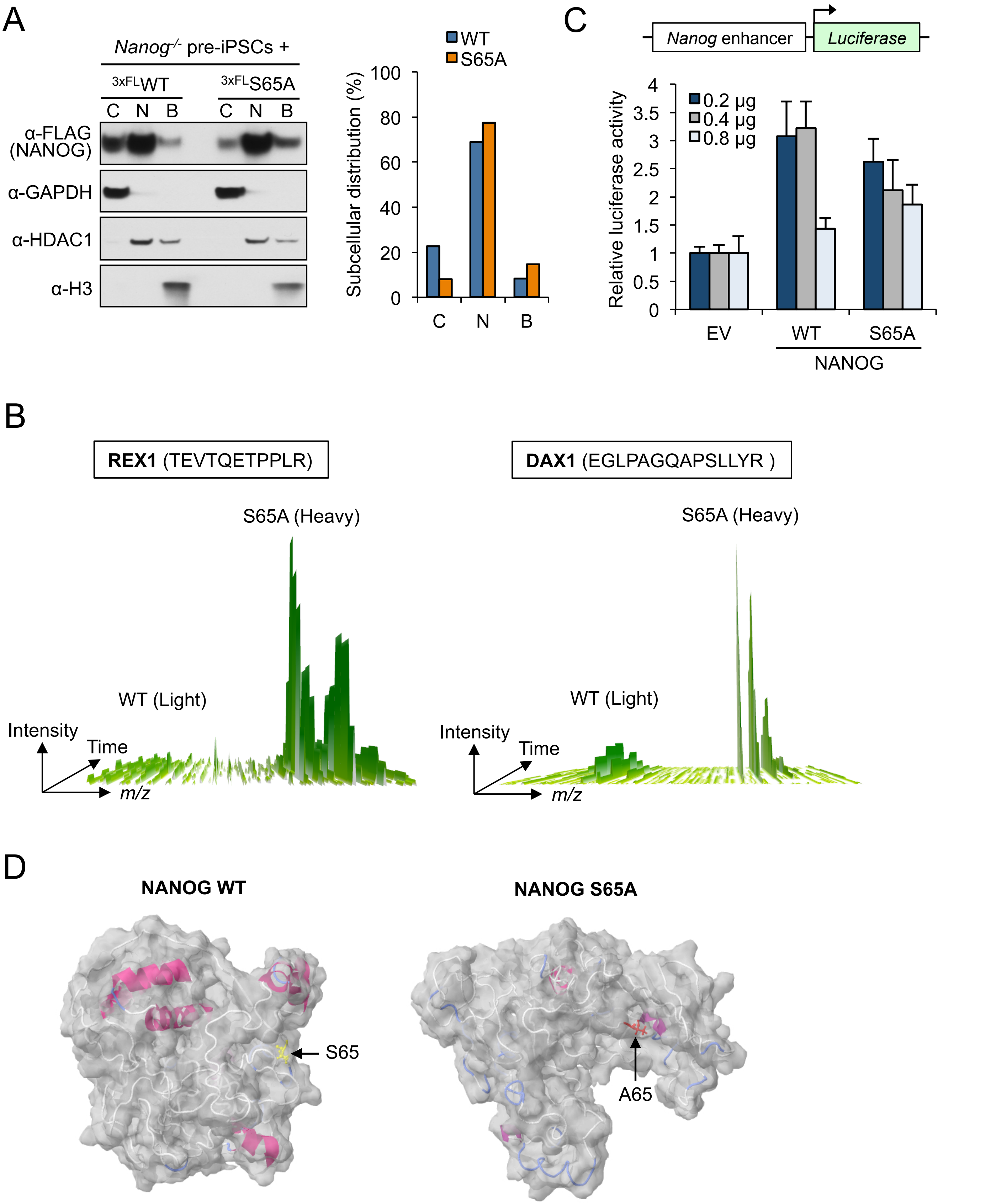
A
Nanog-/- pre-iPSCs +
3xFLWT
3xFLS65A
C N B
C N B
α-FLAG (NANOG)
α-GAPDH
α-HDAC1
α-H3
WT
S65A
Subcellular distribution (%)
0
20
40
60
80
100
C
N
B
C
Nanog enhancer
Luciferase
0.2 μg
0.4 μg
0.8 μg
Relative luciferase activity
0
0.5
1
1.5
2
2.5
3
3.5
4
EV
WT
S65A
NANOG
B
REX1 (TEVTQETPPLR)
DAX1 (EGLPAGQAPSLLYR)
S65A (Heavy)
WT (Light)
Intensity
Time
m/z
S65A (Heavy)
WT (Light)
Intensity
Time
m/z
D
NANOG WT
NANOG S65A
S65
A65

Figure S2

SUPPLEMENTAL TABLES

Table S1. List of qRT-PCR primers used in this study:

Gene	Forward	Reverse
Esrrb	CAGGCAAGGATGACAGACG	GAGACAGCACGAAGGACTGC
Oct4 (Endo.)	TCTTTCCACCAGGCCCCCGGCTC	TGCGGGCGGACATGGGGAGATCC
Sall4	AATGCTGTGCCGAGTTCTTT	GTGCCCAGCTTCTTCAAGTC
Dax1	ATCTGGAAGCAGGGCAAGTA	TCCTGTACCGCAGCTATGTG
Tet1	ATTGAGGTGGAGAAGTGGG	GGAGAAGGGTTGGTTTGC
β-actin	CACAGCTTCTTTGCAGCTCCTT	CGTCATCCATGGCGAACTG

SUPPLEMENTAL EXPERIMENTAL PROCEDURES

ESC culture and LIF withdrawal colony formation assays

Nanog conditional knockout (NgcKO) mESCs were generated as described (Das et al., 2011) and were maintained on gelatin-coated cell culture dishes in standard mESC ("serum + LIF") culture medium [DMEM high glucose (Gibco), 15% heat inactivated FBS (Corning), Pen/Strep (Gibco), Nucleoside mix (Sigma), L-glutamine (Gibco), MEM Non-essential amino acids (Gibco), β-mercaptoethanol (0.1 mM), and recombinant LIF (20 ng/mL)]. ESCs were stably transfected with PB transposon vectors along with PB transposase (PBase) in a 1:2 PB:PBase ratio. Transfections for pre-iPSCs were performed in suspension using 1 µL of Lipofectamine 2000 transfection reagent (Invitrogen) per µg of transfected DNA. 24 hours after transfection, stable line selection began with 10-20 µg/mL Blasticidin S for 5-7 days, with passaging of cells at least once during drug selection.

For LIF withdrawal colony formation assays, NgcKO ESCs were seeded in serum + LIF at a density of 1000 cells per well of a 6-well gelatin-coated plate and allowed to attach for 24 hours. The next day, the culture medium was switched to standard mESC medium without LIF plus 1 µg/mL of doxycycline (Dox). This "Serum – LIF + Dox" medium was changed daily with fresh Dox. On day 5 or 6, plates were stained for alkaline phosphatase (AP) activity (Sigma) and colonies were counted under brightfield microscopy.

IP-MS identification of endogenous NANOG phosphorylation sites

J1 mouse ESCs were expanded to 4 15-cm gelatin-coated cell culture dishes in standard serum + LIF medium supplemented with 1x PhosSTOP phosphatase inhibitor cocktail (Roche, 04906845001). Cells were trypsinized, pelleted, and washed briefly with cold PBS. Pellets were resuspended twice in cold Nuclear Extract Buffer A [10 mM HEPES (pH 8.0), 1.5 mM $MgCl_2$, 10 mM KCl] supplemented with 0.2 mM PMSF, 1x protease inhibitor cocktail (Sigma, P8340), and 1x PhosSTOP to eliminate cytoplasmic lysates. The remaining nuclear pellets were then resuspended in Lysis Buffer (same as above for western blotting) to extract nuclear lysates, and cell membranes and debris were removed by centrifugation for 15 min at 21,000 x g at 4 °C. The salt concentration of nuclear extracts was then increased to 500 mM NaCl to remove any contaminating NANOG interacting partners.

One hundred and fifty microliter (µL) of Protein G agarose (Roche, 11243233001) beads were equilibrated in BSA Buffer [1% BSA in PBS] and then rotated overnight at 4 °C with 40 µg of α-NANOG antibody (Bethyl, A300-397A-2). The next day, nuclear extracts were added to the bead/antibody mixture and rotated for 3 hours at 4 °C. Beads were then washed 4 times in Wash Buffer [250 mM NaCl, 20 mM Tris (pH 7.6), 20% glycerol, 0.05% NP-40, 0.2 mM EDTA, 0.2 mM PMSF], and protein was eluted twice from the beads by adding 4x SDS-DTT elution buffer and boiling at 95 °C for 5 minutes each time. Eluted protein was then concentrated using Amicon Ultra 3K centrifugal filters (Millipore) and subjected to SDS-PAGE. Finally, whole lanes were excised from the gel and subjected to LC-MS/MS mass spectrometry analysis.

Samples were reconstituted in 5-10 µL of HPLC solvent A (2.5% acetonitrile, 0.1% formic acid). A nano-scale reverse-phase HPLC capillary column was created by packing 5 µm C18 spherical silica beads into a fused silica capillary (100 µm inner diameter x 12 cm length) with a flame-draw tip. After equilibrating the column, each sample was loaded onto the column. A gradient of acetonitrile from 2.5% to 97.5% was used to elute the peptides. As peptides eluted, they were subjected to electrospray ionization and then entered into an LTQ-Orbitrap-Velos mass spectrometer (Thermo Finnigan) with collision-induced dissociation (CID). Eluting peptide were detected, isolated, and fragmented to produce a tandem mass spectrum of specific fragment ions for each peptide. MS data were processed by Thermo Proteome Discoverer software with SEQUEST engine against Swiss-Prot mouse protein sequence database. All NANOG phosphorylation sites were confirmed by manual annotation of the MS/MS spectrum. Information of the fragment ions refers to the MS-product utility at UCSF Proteinprospector (http://prospector.ucsf.edu).

Pre-iPSC reprogramming

Nanog$^{-/-}$ neural stem cell (NSC)-derived pre-iPSCs were generated and used for reprogramming as described (Silva et al., 2009). Briefly, pre-iPSCs maintained in serum + LIF culture medium on gelatinized cell culture dishes, and were stably transfected with combinations of PiggyBac-CAG (PB) transposon vectors along with PB transposase (PBase) in a 1:2 PB:PBase ratio. Transfections and Blasticidin selection of pre-iPSCs were performed exactly as described above for NgcKO ESCs.

For pre-iPSC reprogramming, 1.0 x 10^4 pre-iPSCs were seeded after selection onto gelatin-coated 12-well plates on top of a feeder layer of irradiated mouse embryonic fibroblasts (MEFs) (1.0 x 10^6 MEFs per 12-well plate) and grown in serum + LIF for 2 days before medium switch to serum-free, N2B27 medium [DMEM/F12 (Gibco) and Neurobasal (Gibco) media were used in a 1:1 ratio, N2 supplement (Gibco), B27 supplement (Gibco), L-glutamine (Gibco), Pen/Strep (Gibco), and β-mercaptoethanol (0.1 mM)] supplemented with recombinant LIF (20 ng/mL), MEK inhibitor (PD325901; 1 μM final), and GSK3β inhibitor (CHIR99021; 3 μM final) ("2i + LIF" medium). Beginning on day 6 of reprogramming, 200 μg/mL G418 was added to 2i + LIF medium to positively select for activation of endogenous *Nanog* elements (Chambers et al., 2007). On day 10 in 2i + LIF, plates were stained for AP activity and iPSC colonies were counted under brightfield microscopy.

Protein kinase C epsilon (PKCε) inhibition during pre-iPSCs reprogramming was performed using 10 μM PKCε peptide inhibitor (Santa Cruz, sc-3095) or 10 μM scrambled peptide negative control (Santa Cruz, sc-3100). Pan-PKC inhibition was performed using 5 μM Go6983 (Tocris, 2285) or DMSO vehicle control. All inhibitors were added on day 0 of reprogramming in 2i + LIF.

MEF reprogramming

MEF reprogramming was performed as described (Vidal et al., 2014) with some modifications. Briefly, 3.0 x 10^4 reprogrammable MEFs containing a Dox-inducible OKSM cassette were infected with retroviral NANOG WT, NANOG S65A, or EV control. The next day, 2000 infected MEFs/well were seeded on top of a feeder layer of irradiated mouse embryonic fibroblast feeders on a 6-well plate coated with gelatin, in "Dox + 3c"-containing ESC medium. On day 6, medium was switched to ESC medium without Dox or 3c, and plates were stained for alkaline phosphatase activity on day 10.

SILAC IP-MS to compare NANOG WT and S65A interactomes in pre-iPSCs

SILAC IP-MS was performed as described (Ding et al., 2015) with some modifications. Briefly, *Nanog*$^{-/-}$ pre-iPSCs expressing ^{3xFLAG}NANOG WT or ^{3xFLAG}NANOG S65A were each expanded to 8 15-cm dishes after culturing for 2 weeks in SILAC ESC medium [SILAC DMEM (Thermo Scientific, 89985), 10% dialyzed SILAC FBS (Thermo Scientific, 88440), Pen/Strep (Gibco), Nucleoside mix (Sigma), L-glutamine (Gibco), MEM Non-essential amino acids (Gibco), β-mercaptoethanol (0.1 mM), and recombinant LIF (20 ng/mL)] supplemented with either light or heavy lysine and arginine. Pre-iPSC nuclear extracts containing either ^{3xFLAG}NANOG WT or ^{3xFLAG}NANOG S65A were pre-cleared with 100 μL of Protein G agarose beads (Roche, cat.# 11243233001) rotating overnight at 4 °C. The next day, ^{3xFLAG}NANOG WT or ^{3xFLAG}NANOG S65A were immunoprecipitated using 200 μL of EZview red α-FLAG M2 affinity gel beads (Sigma), and were rotated at 4 °C for 3 hours. After washing beads, ^{3xFLAG}NANOG WT or ^{3xFLAG}NANOG S65A were eluted from α-FLAG beads with a 3xFLAG peptide (Sigma, F4799) solution (0.5 mg/mL). Eluted protein was then concentrated using Amicon Ultra 3K centrifugal filters (Millipore), quantified, mixed in a 1:1 ratio for each sample, and then subjected to SDS-PAGE. Finally, whole lanes were excised from the gel and subjected to quantitative LC-MS/MS mass spectrometry analysis.

LC-MS/MS analysis was performed as described earlier for NANOG phospho site mapping. MS data were processed by MaxQuant software with Andromeda search engine (http://www.maxquant.org). Spectrum was search against the Swiss-Prot mouse protein sequence database. All parameters followed the default setting. A cutoff of 2-fold change was applied for the normalized H/L ratios for the quantified proteins.

Structural prediction and modeling of NANOG WT and S65A

The full-length amino acid sequences for NANOG WT or NANOG S65A were submitted in FASTA format to the I-TASSER online platform for structural prediction (http://zhanglab.ccmb.med.umich.edu/I-TASSER/). For each submission, I-TASSER outputted the top 5 structural prediction models in PDB file format, which were quantitatively scored on confidence (C-score) based on threading template alignments and structural assembly simulations. Predicted structures for NANOG WT and NANOG S65A with the highest C-scores (indicative of highest confidence) were selected from their respective top 5 models. PDB files for NANOG WT and NANOG S65A were then manipulated in Jmol Viewer to generate the models presented.

Western blotting

Whole cell lysates from stable NgcKO ESCs and *Nanog*$^{-/-}$ pre-iPSCs were harvested using Lysis Buffer [50 mM HEPES (pH 7.6), 250 mM NaCl, 0.1% NP-40, 0.2 mM EDTA, 1.4 mM β-mercaptoethanol, 0.2 mM PMSF, 1x

protease inhibitor cocktail (Sigma, P8340)]. Lysates were subjected to SDS-PAGE using Mini-PROTEAN precast TGX 4-20% polyacrylamide gels (Bio-Rad). After transfer, membranes were blocked with 5% milk in TBS + 0.1% Tween, and then blotted with the following primary antibodies: α-FLAG M2 (Sigma, F3165), α-GAPDH (ProteinTech, 10494-1-AP), α-NANOG (Bethyl, A300-397A-2), α-VINCULIN (Abcam, ab129002), α-HDAC1 (Bethyl, A300-713A), α-HISTONE 3 (H3) (Abcam, ab1791), α-DAX1 (Santa Cruz, sc-841, K-17), α-OCT4 (Santa Cruz, sc-5279, C-10), α-SALL4 (Santa Cruz, sc-101147, EE-30), α-TET1 (Millipore, 09-872), α-ESRRB (Abcam, ab12986), and the following secondary antibodies: donkey α-mouse IgG-HRP (Santa Cruz, sc-2096), goat α-rabbit IgG-HRP (Santa Cruz, sc-2004). Western blot bands were quantified using ImageJ64 software by determining the FLAG/VINCULIN signal ratio for each sample. These ratios were then normalized to NANOG WT levels, and were set to 1.

Multiple sequence alignment of mammalian NANOG proteins

FASTA sequences for mouse (*Mus musculus*), human (*Homo sapiens*), chimpanzee (*Pan troglodytes*), bovine (*Bos taurus*), rat (*Rattus norvegicus*), macaque (*Macaca fascicularis*), and pig (*Sus scrofa*) NANOG N-terminal domains were obtained from UniProt (www.uniprot.org) and were aligned using the Clustal Omega online tool (www.ebi.ac.uk/Tools/msa/clustalo/).

Statistical analysis

Statistical analyses for all reprogramming experiments and ESC colony formation assays were performed using an unpaired, two-tailed Student's t-test, with significance values indicated in the figure legends. All error bars throughout the figures represent standard deviation (SD).

Protein stability measurement

Pre-iPSCs expressing ^{3xFLAG}NANOG WT or ^{3xFLAG}NANOG S65A were treated with 50 μg/mL of cycloheximide (Sigma, C4859) over a 6-hour time course. Whole cell lysates were extracted and subjected to western blotting as described above. Western blot bands were also quantified as described above, and were then plotted normalized to untreated (0 hours) samples, set to 100%. Protein half-lives of NANOG WT and NANOG S65A were calculated using linear regression analysis in Microsoft Excel to determine the time at which 50% of each protein was remaining.

Immunofluorescence

Immunofluorescence on pre-iPSCs expressing ^{3xFLAG}NANOG WT or ^{3xFLAG}NANOG S65A was performed as described (Gingold et al., 2014). Briefly, pre-iPSCs were cultured on glass slides, fixed in 4% paraformaldehyde, washed with PBS, and then permeabilized with 0.25% Triton X-100. Next, fixed cells were blocked with 10% BSA in PBS and then incubated overnight at 4 °C with the following primary antibodies: α-FLAG M2 (Sigma, F3165), α-GAPDH (ProteinTech, 10494-1-AP), and the following secondary antibodies: donkey anti-mouse AlexaFluor 488, donkey anti-rabbit RRX. DAPI was mixed with secondary antibodies as a nuclear marker. Fixed cells were then washed 3x in PBS and mounted for imaging by fluorescence microscopy.

Luciferase assays

Nanog enhancer-driven luciferase assays were performed as described (Ding et al., 2015) using 0.2 μg, 0.4 μg, and 0.8 μg of PB-empty vector (EV), NANOG WT, or NANOG S65A plasmids in HEK 293T cells.

qRT-PCR

Total RNA was extracted using the RNeasy kit (Qiagen) and cDNA was generated using qScript (Quanta, cat. # 95048). Relative expression levels were determined using the LightCycler 480 SYBR Green I Master mix (Roche, cat. # 4729749001), and expression levels were normalized to β-actin. qRT-PCR experiments were performed in triplicate on a LightCycler Real Time PCR System (Roche). See Table S1 for a list of all primers used in this study.

SUPPLEMENTAL REFERENCES

Chambers, I., Silva, J., Colby, D., Nichols, J., Nijmeijer, B., Robertson, M., Vrana, J., Jones, K., Grotewold, L., Smith, A., 2007. Nanog safeguards pluripotency and mediates germline development. Nature 450, 1230–1234. doi:10.1038/nature06403

Gingold, J.A., Fidalgo, M., Guallar, D., Lau, Z., Sun, Z., Zhou, H., Faiola, F., Huang, X., Lee, D.-F., Waghray, A., Schaniel, C., Felsenfeld, D.P., Lemischka, I.R., Wang, J., 2014. A genome-wide RNAi screen identifies opposing functions of Snai1 and Snai2 on the Nanog dependency in reprogramming. Molecular Cell 56, 140–152. doi:10.1016/j.molcel.2014.08.014
